# Associations Among PCSK9 Levels, Atherosclerosis-Derived Extracellular Vesicles, and Their miRNA Content in Adults With Obesity

**DOI:** 10.3389/fcvm.2021.785250

**Published:** 2022-01-07

**Authors:** Chiara Macchi, Maria Francesca Greco, Chiara Favero, Laura Dioni, Laura Cantone, Mirjam Hoxha, Luisella Vigna, Giulia Solazzo, Alberto Corsini, Maciej Banach, Angela C. Pesatori, Valentina Bollati, Massimiliano Ruscica

**Affiliations:** ^1^Department of Pharmacological and Biomolecular Sciences, Università degli Studi di Milano, Milan, Italy; ^2^Epidemiology, Epigenetics and Toxicology (EPIGET) Department of Clinical Sciences and Community Health, Università degli Studi di Milano, Milan, Italy; ^3^Occupational Medicine Unit, Fondazione Cà Granda, Istituto di Ricovero e Cura a Carattere Scientifico (IRCCS) Ospedale Maggiore Policlinico, Milan, Italy; ^4^Istituto di Ricovero e Cura a Carattere ScientificoI (RCCS) Multimedica, Milan, Italy; ^5^Department of Preventive Cardiology and Lipidology, Medical University of Lodz, Lodz, Poland; ^6^Cardiovascular Research Centre, University of Zielona Gora, Zielona Gora, Poland

**Keywords:** atherosclerosis, PCSK9 (proprotein convertase subtilisin kexin type 9), extracellular vesicles (EVs), miR-150, obesity, low-density lipoprotein receptor (LDLR)

## Abstract

**Background:** Extracellular vesicles (EV) concentration is generally increased in patients with cardiovascular diseases, although the protective role of EVs in atherosclerosis has been reported. Among the specific cargo of EVs, miRNAs contribute to different stages of atherosclerosis. Aim of the present report has been to investigate, in individuals with obesity, the interplay among EVs derived from cells relevant for the atherosclerotic process (i.e., platelets, endothelium, monocytes/macrophages, and neutrophils), their miRNA content and proprotein convertase subtilisin/kexin type 9 (PCSK9), one of the main regulators of low-density lipoprotein receptor (LDLR).

**Methods and Results:** EVs have been isolated from 936 individuals with obesity (body mass index = 33.6 ± 5.6 Kg/m^2^) and a raised cardiovascular risk (e.g., LDL-C = 131.6 ± 36.4 mg/dL, HOMA-IR = 3.1, and roughly 50% on anti-hypertensive medications). PCSK9 levels were negatively associated with EV count in the range 150–400 nm and with those derived from macrophages (CD14^+^), endothelium (CD105^+^), and neutrophils (CD66^+^). The association between PCSK9 and platelet-derived EVs (CD61^+^) was modified by platelet counts. PCSK9 was significantly associated with five EV-derived miRNAs (hsa-miRNA−362−5p,−150,−1244,−520b-3p,−638). Toll-like receptor 4 and estrogen receptor 1 were targeted by all five miRNAs and LDLR by four. The effect on LDLR expression is mainly driven by hsa-miR-150. Considering the implication of EV in atherosclerosis onset and progression, our findings show a potential role of PCSK9 to regulate EV-derived miRNAs, especially those involved in inflammation and expression of low-density lipoprotein receptor (LDLR) receptor.

## Introduction

Atherosclerosis is a leading cause of morbidity and mortality worldwide (1). Vascular diseases associated with atherosclerosis include ischemic heart disease, ischemic stroke, and peripheral artery disease. Atherosclerosis is a complex disorder, and many components of the vascular, metabolic, and immune systems are involved in this process. It is a progressive inflammatory disease affecting large and medium-sized blood vessels by triggering the formation of a plaque in the vessel wall (2). Increasing evidence is emerging on the possible role of extracellular vesicles (EVs), which are powerful mediators of inter-cellular and inter-system communication. Extracellular vesicles are membrane-bound particles released by cells in biological fluids, both in physiological and pathological contexts. Although it is generally thought that EV concentration is increased in patients with cardiovascular diseases (3, 4), and a large amount of EVs has been observed within the atherosclerotic plaque (5), the protective role of EVs in atherosclerosis has been also reported (6). Indeed, the specific cargo of EVs, composed of microRNAs (miRNAs), mRNAs, proteins, lipids, and organelles varies according to environmental stimuli (7), risk factors (8, 9), and even in each stage of the atherosclerotic process (10). miRNAs, in particular, contribute to the different stages of atherosclerosis, including inflammation, endothelial dysfunction, atherogenesis and angiogenesis, atherosclerotic plaque progression, and rupture (11).

To complement the possible role of EVs and their miRNA content in modulating these processes, the impact of proprotein convertase subtilisin/kexin type 9 (PCSK9), one of the key regulators of low-density lipoprotein cholesterol (LDL-C), on the atherogenic process cannot be overlooked. Briefly, PCSK9 is expressed in endothelial cells, and at a low level, in macrophages, implying a potential role of PCSK9 in atherosclerosis plaque development (12). PCSK9 can directly increase atherosclerotic lesion inflammation and can promote plaque monocyte infiltration and macrophage inflammation (13). In plaque dendritic cells, PCSK9 regulates the expression of miR-27a, which is involved in lipid metabolism (14).

Obesity is one of the major risk factors for atherosclerosis, even after accounting for other known risk factors, such as hypertension, dyslipidemia, and smoking habit (15). The Global Burden of Disease panel has reported an increase in the burden of elevated body mass index (BMI), with high BMI accounting for 4.0 million deaths in 2015, more than two-thirds of which were caused by cardiovascular diseases (16).

Considering that EVs are vehicles of bioactive molecules (e.g., miRNA) which influence the intertwined relationship among players of the atherosclerotic process, the present study aims to investigate the interplay among PCSK9, EVs derived from cells relevant for the atherosclerotic process (i.e., platelets, endothelium, monocytes/macrophages, and neutrophils) and their miRNA content in a population of subjects with obesity. The identification of plasma molecules that serve as prognostic and diagnostic biomarkers for metabolic diseases and their pathological complications remains challenging because blood-based biomarkers, such as glucose or lipids provide little insight into the tissue types and organs afflicted by metabolic dysfunction.

## Methods

### Study Design and Participants

Nine hundred and thirty-six individuals of the cross-sectional Susceptibility to Particle Health Effects, miRNAs and Exosomes (SPHERE) study were randomly selected (17). These individuals were recruited at the Center for Obesity and Work-Activity (Fondazione IRCCS Ca' Granda Ospedale Maggiore Policlinico in Milan, Lombardy, Italy). The eligibility criteria of the SPHERE study were: (a) older than 18 years at enrolment; (b) overweight/obese according to body mass index (BMI): overweight, BMI between 25 and 30 kg/m^2^; obese: BMI of 30 kg/m^2^ or more; (c) resident in the Lombardy Region at the time of recruitment. Exclusion criteria were: previous diagnosis of cancer, heart diseases, stroke, other chronic diseases, or known diagnosis of diabetes. The study was conformed to the Declaration of Helsinki and each participant provided written informed consent which was approved by the Ethics Committee of Fondazione IRCCS Cà Granda Ospedale Maggiore Policlinico (approval number 1425). Fasting blood drawing was all performed at 9 a.m. in order to avoid circadian variations and processed within 2 h. Blood was collected in two EDTA vacutainers (7.5 ml) and in one PAX gene (2.5 ml). See STROBE statement.

### Clinical and Laboratory Measurements

Body weight and height were determined on a standard scale; body mass index (BMI) and weight-to-height ratio were also calculated. Systolic and diastolic blood pressures (SBP and DBP, respectively) were taken on the left arm using a mercury sphygmomanometer (mean of two measurements taken after 5 min of rest). Plasma lipids/lipoproteins and glucose were determined by established methodologies, as carried out in the laboratory of the Institution. C-reactive protein (CRP) and liver function tests (ALT, AST, and GGT), as well as a full hematological profile (red blood cells, hematocrit, and leukocyte formula), were determined. HbA1c was measured by ion-exchange high-performance liquid chromatography on a VARIANT II Turbo Instrument (Glyco Hb Control, Menarini Diagnostics, Firenze, Italy); insulin by electrochemiluminescence immunoassay (ECLIA) on the Modular P automated analyzer (Roche, Basel, Switzerland). Homeostasis model assessment-insulin resistance (HOMA-IR) was computed as fasting plasma glucose (mg/dL) times fasting serum insulin (mU/L) divided by 405; quantitative insulin sensitivity check index (QUICKI) was given by 1/[Log (Fasting Insulin, μU/mL) + Log (Fasting Glucose, mg/dL)]. Fifty microliter of plasma were used to quantify a custom panel of cytokines by the Luminex xMAP^®^-based technology (MYRIAD RBM, Inc., Austin, TX), a multiplex immunoassay simultaneously quantifying multiple protein analytes in a single run. Interferon-γ, interleukin (IL)-8, IL-10, IL-18, macrophage inflammatory protein-1α (CCL3), macrophage inflammatory protein-1β (CCL4), monocyte chemoattractant protein-1 (CCL2), and tumor necrosis factor-α (TNF-α) were measured. When the concentration was below the lower limit of quantification (LLOQ), data were replaced by half of the lower limit of quantification (LLOQ/2).

### Enzyme-Linked Immunosorbent Assay

Plasma PCSK9 concentrations were measured by a commercial ELISA kit (R&D Systems, MN). Samples were diluted at 1:20 and incubated onto a microplate pre-coated with a monoclonal human-PCSK9-specific antibody. Sample concentrations were obtained by a four-parameter logistic curve-fit, with a minimum detectable PCSK9 concentration of 0.219 ng/mL (18).

### EVs Isolation

Isolation, purification, and characterization of EVs were performed by following MISEV 2018 Guidelines (19). Briefly, EDTA-blood was centrifuged 1,200 × g for 15 min at room temperature to obtain platelet-free blood plasma. Plasma was further centrifuged at 1,000, 2,000, and 3,000 × g for 15 min at 4°C, discarding the pellet to clean the cell debris. To prepare EV pellet for Nanosight and Flow Cytometry, 1.5 mL of fresh plasma was transferred into an ultracentrifuge tube (Quick-Seal^®^-Round-Top, Polypropylene, 13.5 mL-Beckman Coulter, Inc.) and filled up with PBS, filtered with 0.10 μm pore size membrane (StericupRVP, 0.10 μm, polyethersulfone filter- Merck Millipore) to minimalize the background contribution of interfering particles. Plasma was then ultracentrifuged (BeckmanCoulter Optima-MAX-XP) at 110,000 x g for 75 min at 4°C, to obtain an extracellular vesicles-rich pellet. The pellet was re-suspended with 500 μL triple 0.10 μm pore size membrane-filtered PBS. To prepare the EV pellet for miRNA extraction, 1.5 mL of fresh plasma was transferred into an ultracentrifuge tube (Centrifuge bottles polycarbonate, 10.4 mL-Beckman Coulter) and filled up with PBS. Plasma was then ultracentrifuged (BeckmanCoulter Optima-MAX-XP) at 110,000 x g for 75 min at 4°C, decanted, and the EV pellet was kept at −80°C until miRNA extraction.

### Nanoparticle Tracking Analysis

The number and dimension of EVs were assessed by nanoparticle tracking analysis (NTA). This technique measures the Brownian motion of vesicles suspended in a fluid and displays them in real-time through a CCD camera with high sensitivity. Using a Nanosight LM10-HS system (NanoSight Ltd., Amesbury, UK), EVs were visualized by laser light scattering, five recordings of 30 s were performed for each sample. Collected data were analyzed with NTA software, which provided high-resolution particle size distribution profiles and concentration measurements of the EVs.

### Flow Cytometry

EVs were characterized by MACSQuant analyzer flow cytometer (Miltenyi Biotec, Bergisch Gladbach, Germany) according to customer protocol for characterization of EVs (http://bit.ly/2sCN9vy). Fluoresbrite^®^ Carboxylate Size Range Kit I (0.2, 0.5, 0.75, and 1 μm), was used to set the calibration gate on MACSQuant analyzer.

To evaluate EV integrity, 60 μL sample aliquots were stained with 0.02 μM 5(6)-carboxyfluorescein diacetate N-succinimidyl ester (CFSE) at 37°C for 20 min in the dark. Each aliquot of CFSE stained sample was than incubated with a specific antibody: CD14-APC (Clone TÜK4), CD105-APC (clone: 43A4E1), CD326 (EpCAM)-APC (clone: HEA-125), CD66abce-FITC (clone: TET2), and CD61-APC (clone: Y2/51). All of them were purchased from Miltenyi Biotec.

Before use, each antibody was centrifuged at 17,000 × g for 30 min at 4°C to eliminate aggregates. The stained PBS control sample was acquired to detect the autofluorescence of the antibody. Quantitative multiparameter analysis of flow cytometry data was carried out using FlowJo Software (Tree Star).

### EVs-miRNA Isolation and Analysis

Isolation of miRNAs from EVs was performed with the combination of miRNeasy kit and RNeasy Cleanup Kit (Qiagen), according to the manufacturer's protocol. EV-miRNAs quality and integrity were assessed through the “2100 Bioanalyzer RNA system” (Agilent Technologies). MiRNAs were eluted in 20 μL of Nuclease-Free Water and stored at −80°C, until to use. MiRNAs reverse transcription (RT) and preamplification reactions, followed by real-time RT-PCR analysis with the QuantStudio™ 12K Flex OpenArray^®^ Platform (Applied Biosystem), were previously described (20). Gene Expression Suite Software (Applied Biosystem) was used to process miRNA expression data from the “TaqMan™ OpenArray™ Human MicroRNA panel” (ThermoFisher) analysis.

### Total RNA Extraction From PAXgene Blood and LDLR Expression

Total RNA was extracted from each PAXgene using MirVana RNA isolation kit (ThermoFisher), following manufacturer's instructions. RNA was eluted in 80 μL of Nuclease-Free Water, total yields and quality were finally assessed by Thermo Scientific™ NanoDrop. The cDNA synthesis reaction was performed with Maxima First Strand cDNA Synthesis kit (ThermoFisher), following manufacturer's instruction, in a final volume of 15 μl, using 380 ng totalRNA for each sample. qPCR was then performed by using the SYBR™ Select Master Mix (ThermoFisher) and specific primers for LDL receptor (LDLR) and 36B4 as endogenous control. Primers sequences were: forward LDLR (5′ TCTATGGAAGAACTGGCGGC 3′) and reverse LDLR (5′ ACCATCTGTCTCGAGGGGTA); 36B4 forward (5′ CCACGCTGCTGAACATGC 3′); and 36B4 reverse (5′ TCGAACACCTGCTGATGAC 3′). The analyses were performed in triplicate in a 10 μl final volume, with QuantStudio™ 12K Flex in a 384-plate. PCR cycling conditions were as follows: 95°C for 2 min, 40 cycles at 95°C for 15 s, and 60°C for 60 s. Gene Expression Suite Software (Applied Biosystem) was used to process expression data.

### Identification of miRNA Targets Related to Atherosclerosis

To elucidate the possible mechanisms connecting EVs-miRNA content and the biological processes related to atherosclerosis a three-step analysis has been performed. First, Disgenet2r R package was used to find genes related to atherosclerosis (21). Second, a miRNA-target interactions analysis was performed on miRWalk for each miRNA associated with PCSK9. As *bona-fide* miRNA-target interactions was considered only those predicted by at least 2 algorithms among miRWalk, RNA22, miRanda, and Targetscan (in the version provided by miRWalk 2.0) (22). Finally, the miRNA-target interactions analysis data were imported on R software and the genes matching with atherosclerosis-related genes were selected. The gene network and the Venn Diagram were drawn by using R software and GIMP-2.10.

### Statistical Analysis

Descriptive statistics were performed on all variables. Continuous data were expressed as the mean ± standard deviation (SD) or as the median and interquartile range (Q1–Q3), as appropriate. Categorical data were presented as frequencies and percentages. We applied multivariable negative binomial regression models for over-dispersed count observations to evaluate the relationship between circulating PCSK9 levels and EV count (total count from NTA, CD66^+^, CD14^+^, CD61^+^, CD105^+^, and EPCAM^+^). We tested the presence of over-dispersion basing upon the Lagrange Multiplier (LM) test. The regression models were adjusted for age, gender, BMI, smoking habit, use of statin medication, particulate matter (PM)_10_, and apparent temperature measured the day before the blood draw. In the model with the dependent variable CD61^+^ EVs, we adjusted also for platelets concentration, in the CD14^+^ EVs model we adjusted also for monocytes percentage, and in the CD66^+^ EVs model we corrected also for neutrophils percentage. Estimated effects were described as a percentage of variation associated with an increase of 10 ng/mL in PCSK9 concentration. The percentage of variation was defined as (1-incidence rate ratio (IRR))^*^100.

For each EV size, we estimated IRR and 95% CI of total count EV for each 10 ng/mL increment in PCSK9 levels, with negative binomial regression models. Due to the high number of comparisons, we used a multiple comparison method based on Benjamini–Hochberg False Discovery Rate (FDR) to calculate the FDR *P*-value. To display the results of the analyses we used a series graph for IRR and 95% CI and vertical bar charts to represent FDR *P*-values and *P*-values. For the two graphs, X-axis reported the size of EVs (30 to 700 nm). We applied multivariable negative binomial regression models for over-dispersed count observations to evaluate the relationship between circulating PCSK9 levels and EV count. We tested over-dispersion by the likelihood ratio test, and based on its results, we decided to apply the negative binomial regression model.

To examine the potential effect modification of cell percentages and cytokines on EV concentration, we tested the interaction term PCSK9^*^cell percentage/cytokine to the multivariable selected model. The reported results are the ones showing a statistically significant interaction: (a) effect of PCSK9 on CD61^+^ EVs concentration depending on platelets levels; (b) effect of PCSK9 on CD14^+^ EVs concentration depending on monocyte percentage; (c) effect of PCSK9 on EPCAM^+^ and CD105^+^ EVs depending on IL-8 plasmatic concentration.

Multivariable linear regression models were applied to verify the association between PCSK9 and miRNA expression. miRNA expression values were log2-transformed to achieve a normal distribution. The regression models were adjusted for age, gender, BMI, smoking habit, use of statin medication, PM_10_, and apparent temperature measured the day before the blood draw. Due to the high number of comparisons, we applied multiple comparison correction methods based on the Benjamini-Hochberg False Discovery Rate (FDR) to calculate the FDR *P*-value. A volcano plot of Δ% vs. –log10 *P*-values was used to display results. We verified the association between miRNA expression and LDLR with multivariable linear regression models adjusted for age, gender, and BMI. Regression coefficients were calculated per 1 standard deviation increase in each predictor variable. All statistical analyses were performed with SAS software (version 9.4; SAS Institute Inc., Cary, North Carolina, USA). A two-sided *P*-value of 0.05 was considered statistically significant.

## Results

### Characteristics of the Study Population

The present study included 936 individuals with obesity (BMI = 33.6 ± 5.6 Kg/m^2^), 24.7% men and 75.3% women with a mean age of 52.4 ± 14 years. An extensive description of study participants is reported in Table 1. Briefly, 47.8% never smoked, 35.4% were former smokers, and 16.0% current smokers. 45.7% of participants were on hypertensive medications and 12.3% of participants were taking statins. Mean values of total cholesterol (TC), LDL-C, and non-high-density cholesterol (non-HDL-C) were in the upper range of normality (TC = 207.4 ± 40.6 mg/dL, LDL-C = 131.6 ± 36.4 mg/dL, and non-HDL-C = 148.4 ± 40.9 mg/dL). HDL-C and TG levels were also in the normal range: 58.5 ± 15.0 and 119.2 ± 76.2 mg/dL, respectively. Glycemia and glycated hemoglobin were in the high range (99.8 ± 22.2 mg/dL and 39.4 ± 8.4 mmol/mol, respectively). PCSK9 levels were normally distributed, with a mean level of 283.3 ± 96.3 ng/mL. Complete blood count formula and percentages are also reported. Since we previously reported the effect of PM_10_ exposure on EVs release, this variable was used as a covariate in the model. Subjects mean exposure in the day before the blood sampling was equal to 37.9 ± 22.2 μg/m^3^ and the apparent temperature was equal to 12.5 ± 8.2°C.

**Table 1:** Demographic and clinical characteristics of the study participants (*N* = 936).

**Characteristics**	
Age, years	52.4 ± 14.0
Gender	
Males	231 (24.7%)
Females	705 (75.3%)
BMI, kg/m^2^	33.3 ± 5.6
Smoking status	
Never smoker	447 (47.8%)
Former smoker	331 (35.4%)
Current smoker	150 (16.0%)
NA	8 (0.8%)
Blood pressure, mmHg	
Sistolic	126.4 ± 14.9
Diastolic	79.1 ± 8.56
Antihypertensive medications
Yes	427 (45.7%)
No	509 (54.3%)
Statin medications	
Yes	115 (12.3%)
No	821 (87.7%)
Total cholesterol, mg/dL	207 ± 40.6
HDL-C, mg/dL	58.5 ± 15
LDL-C, mg/dL	131.6 ± 36.4
non-HDL-C, mg/dL	148.4 ± 40.9
Triglyceride, mg/dL	119 ± 76.2
PCSK9, ng/mL	283.3 ± 96.3
Glucose, mg/dL	99.8 ± 22.2
Glycated hemoglobin, mmol/mol	39.4 ± 8.4
Insulin, U/mL	15.8 ± 14.1
HOMA-IR	3.1 [1.9;4.9]
QUICKI	0.14 ± 0.01
Hemochrome, 10^3^cell/μL	
White blood cells	6.78 ± 1.73
Red blood cells	4.73 ± 0.44
Hemoglobin	13.6 ± 1.4
Hematocrit	40 ± 3.5
Mean Corpuscular Volume	85 ± 6.4
Platelets	252 ± 60.8
Neutrophils, %	58.4 ± 7.8
Eosinophils, %	2.6 ± 1.7
Lymphocytes, %	31.3 ± 7.2
Monocytes, %	7.3 ± 1.8
Basophils, %	0.5 ± 0.3
Granulocytes, %	61.5 ± 7.5

*BMI, body mass index; HDL-C, high-density lipoprotein cholesterol; HOMA-IR, homeostasis model assessment-insulin resistance; LDL-C, low-density lipoprotein cholesterol; PCSK9, proprotein convertase subtilisin/kexin type 9; QUICKI, quantitative insulin-sensitivity check index. For normal distribution, values are expressed as mean ± standard deviation. When not normally distributed, values are expressed as median [Q1; Q3]. Discrete variables are expressed as counts (%). NA, not available*.

### Association Between PCSK9 Levels and EVs

**Figure 1:**
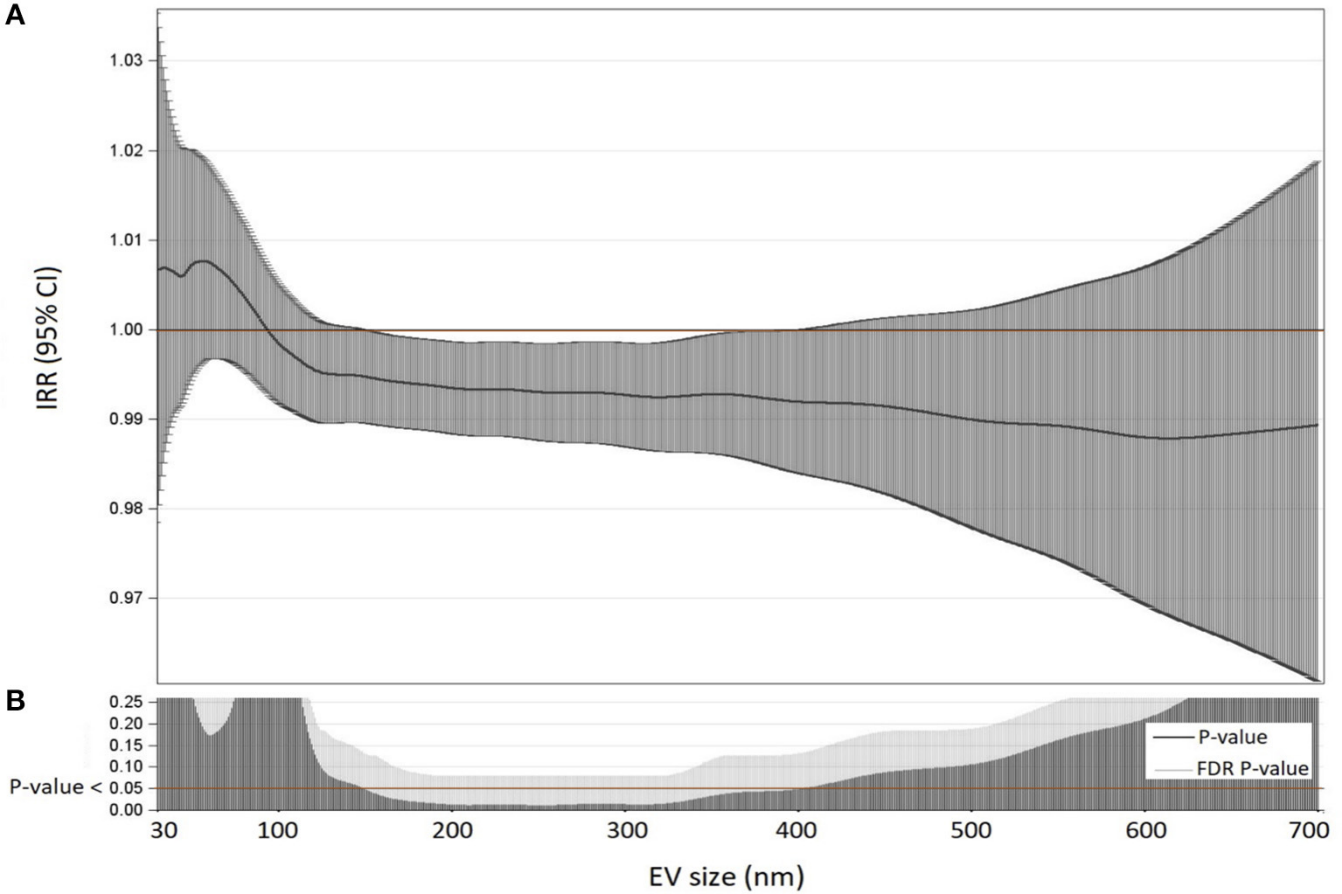
Association between PCSK9 levels and EV concentrations. **(A)** For each EV size (nm) IRR and 95% CI of associations between PCSK9 levels and EV concentrations were reported. IRR represents the percent variation in EVs concentration for 10 ng/mL increase in PCSK9 levels. **(B)** For each size, *P*-value and False Discovery Rate from negative binomial regression models adjusted for age, gender, BMI, smoking habit, statin use, PM_10_, and apparent temperature at the day before the blood draw were reported.

For each subject, the association between PCSK9 levels and EVs count was investigated. Dimension Nanosight analysis showed the plasma EV median concentration was 1,912 x 10^6^/ml of plasma, EVs average size was 212 nm, and EV mode was 155 nm (Supplementary Table 1). Figure 1 describes the Incidence Rate Ratio (IRR) of the association between PCSK9 levels and plasmatic EV concentration. For each EV size (between 30 and 700 nm), the percentage of variation in EV concentration (IRR) associated with every 10 ng/mL increment of PCSK9 levels is reported (Figure 1A). The lower part of the plot (Figure 1B) shows the *P*-values and FDR *p*-values obtained from negative binomial regression models adjusted for age, gender, BMI, smoking habit, statin use, PM_10_, and apparent temperature recorded in the day before the blood draw. The effect of PCSK9 on EV count was negative and significant (*p* < 0.05) for EVs in the range of 150 nm and 400 nm. Taken the different EV sizes as a whole (Table 2), PCSK9 was associated with a decreasing number of EVs (IRR = 0.996; 95% CI 0.992–0.999, *p* = 0.0377).

**Table 2:** Associations among PCSK9 levels (ng/mL) and different classes of EVs evaluated by multivariable negative binomial regression models.

	**IRR**	**95% CI**	***P*** **-value**
EV total count	0.996	0.992–0.999	0.0377
EV _CD14+_ (macrophages/monocytes)	0.990	0.984–0.997	0.0063
EV _CD105+_ (endothelium)	0.994	0.988–0.999	0.0270
EV _CD66+_ (neutrophils)	0.990	0.983–0.996	0.0023
EV _CD61+_ (platelets)	0.999	0.991–1.007	0.8740

*All negative binomial regression models were adjusted for age, gender, BMI, smoking habit, statin use, PM_10_, and apparent temperature at the day before the blood draw*.

*For CD61^+^ EV, the model was also corrected for platelets; for CD66^+^, the model was also adjusted for percentage of neutrophils; for CD14^+^ EV, the model was also adjusted for percentage of monocytes*.

IRR stands for Incidence Rate Ratios and represents the percent decrease [(1 –IRR)^*^100] in EV concentration for 10 ng/mL increase in PCSK9 levels.

*BMI, body mass index; EV, Extracellular vesicles; PM, particulate matter*.

In order to characterize the cellular source of EVs, we analyzed enriched platelet-derived EVs (CD61^+^ EVs), enriched monocyte/macrophage-derived EVs (CD14^+^ EVs), enriched endothelial-derived EVs (CD105^+^ EVs), and enriched neutrophil-derived EVs (CD66^+^ EVs). Table 2 reports the associations between PCSK9 and EVs released from each cell type above described. A significant decrease was observed for CD105^+^ EVs (IRR = 0.994; 95% CI 0.988–0.999, *p* = 0.027), CD14^+^ EVs (IRR = 0.990; 95% CI 0.984–0.997, *p* = 0.0063), and CD66^+^ EVs (IRR = 0.990; 95% CI 0.983–0.996, *p* = 0.0023).

**Figure 2:**
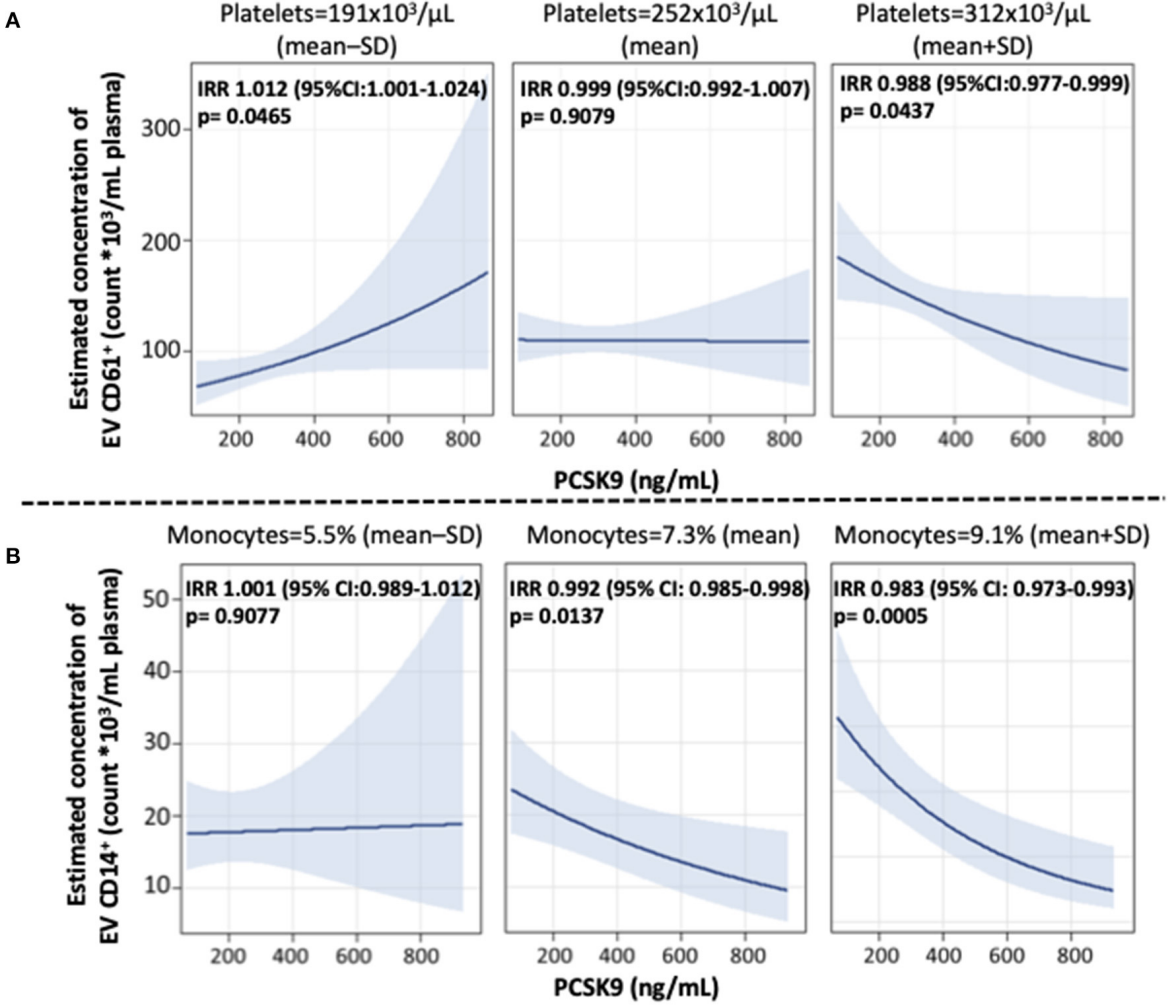
Interaction effect of PCSK9 levels and platelets on EV CD61^+^ concentrations and monocytes on EV CD14^+^ concentrations. **(A)** Strength of association between PCSK9 and EV CD61^+^ at three selected levels of platelets [mean-standard deviation (SD), mean, and mean + SD value]. **(B)** Adjusted incidence rate ratios (IRR) were reported for a 10 ng/mL increase in PCSK9 concentration, at each level of monocytes. The negative binomial regression model was adjusted for age, gender, BMI, smoking habit, statin use, PM_10_, and apparent temperature measured on the day before the blood draw. *P*-value of interaction term PCSK9*CD61^+^ was 0.0166 **(A)** and PCSK9*CD14^+^ was 0.0322 **(B)**. BMI, body mass index; PCSK9, proprotein convertase subtilisin/kexin type 9; PM, particulate matter.

**Figure 3:**
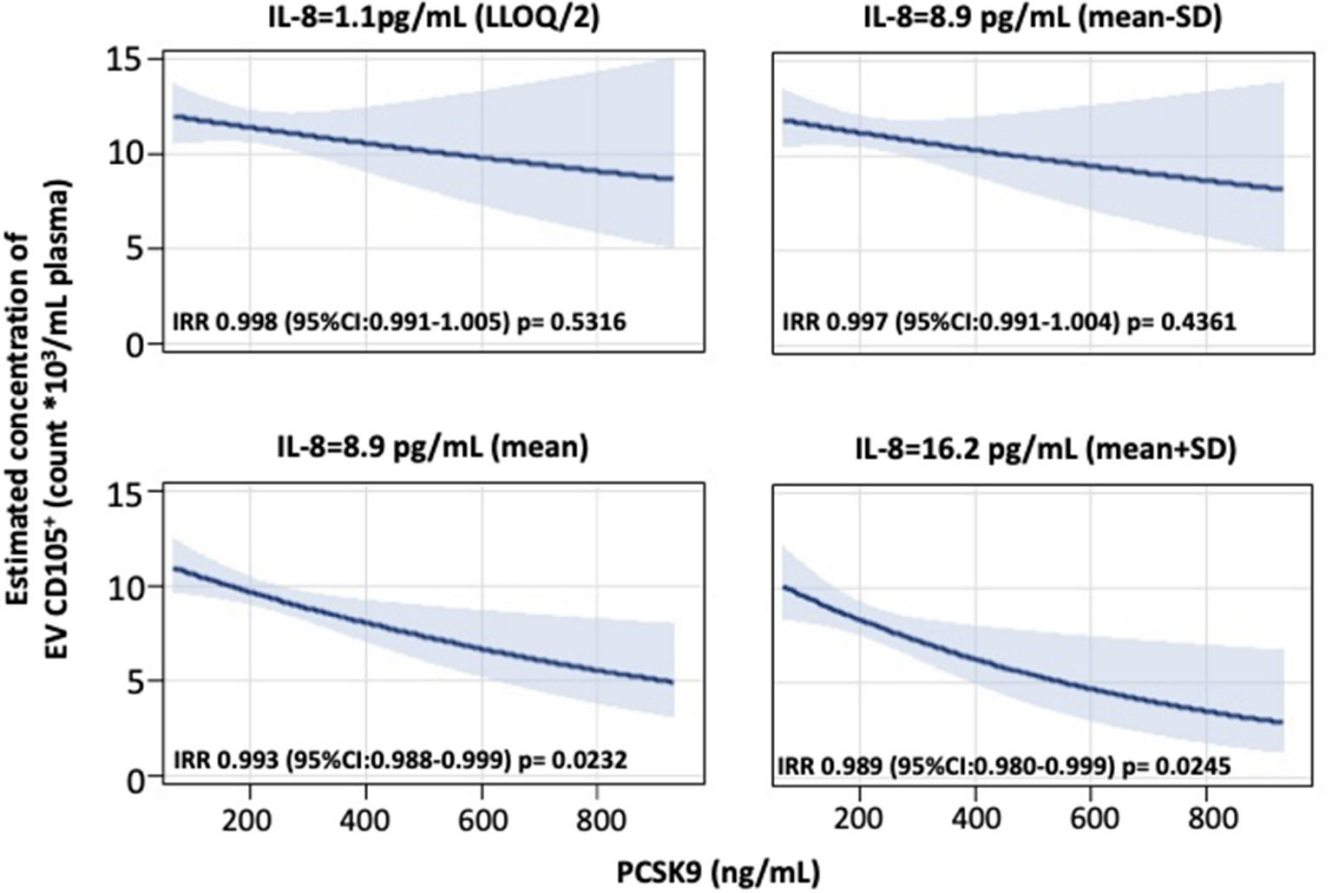
Interaction effect of PCSK9 and IL-8 levels on EV CD105^+^ concentrations. Strength of association between PCSK9 levels and EV CD105^+^ concentrations at four selected levels of IL-8 [lower limit of quantification (LLOQ)/2, mean – standard deviation (SD), mean, and mean + SD value]. *P*-value of interaction term PCSK9*CD105^+^ was 0.1532. Adjusted incidence rate ratios (IRR) were reported for a 10 ng/mL increase in PCSK9 concentration, at each level of IL-8. The negative binomial regression model was adjusted for age, gender, BMI, smoking habit, statin use, PM_10_, and apparent temperature measured on the day before the blood draw. BMI, body mass index; PCSK9, proprotein convertase subtilisin/kexin type 9; PM, particulate matter.

Further, we found that blood cell count and inflammation were modifiers on the above-reported associations. Specifically, the effect of PCSK9 on platelet-derived EVs was modified by platelet count (Figure 2A). While in subjects with a lower platelet count (mean – SD: 191 × 10^3^ platelets/μL), PCSK9 was associated with a rise in the number of CD61^+^ EVs (IRR = 1.012; 95% CI 1.001–1.024, *p* = 0.046), the opposite (IRR = 0.988; 95% CI 0.977–0.999, *p* = 0.043) was found in individuals with a higher platelet count (mean + SD: 312 × 10^3^ platelets/μl). Using a similar statistical approach, the effect of PCSK9 on macrophages/monocytes-derived EVs (CD14^+^) was modified by monocyte count (Figure 2B). The negative effect was mainly driven by subjects with a higher monocyte count (IRR = 0.983; 95% CI 0.973–0.992, *p* = 0.0005). The effect of PCSK9 on endothelial-derived-EVs (CD105^+^) was modified by plasmatic IL-8 (Figure 3), as the effect was larger for subjects with a high concentration of IL-8 (IRR = 0.989; 95% CI 0.980–0.999, *p* = 0.024). Finally, the interaction between PCSK9 and neutrophils-derived-EVs (CD66^+^) was not modified by neutrophils count (*p* = 0.7683).

### Association Between PCSK9 Levels and miRNA Related to Atherosclerosis

To assess the possible role of PCSK9 in modulating miRNA-EV content, an OpenArray technology was used. After data cleaning, 527 miRNAs were expressed in at least one subject. In a model adjusted for age, gender, BMI, smoking habit, use of statin, PM_10_, and apparent temperature measured the day before the blood draw, PCSK9 levels were associated with a significant increase of 35 miRNAs and a significant decrease of 29 miRNAs (Supplementary Figure 1). After FDR adjustment for multiple comparisons (FDR *P* < 0.1), PCSK9 levels were positively associated with hsa-miR-362-5p (Δ% 3.069; 95%CI 1.6, 4.6; *p* = 0.0298), hsa-miR-150 (Δ% 5.238; 95% CI 2.5, 8.0; *p* = 0.0298), and hsa-miR-1244 (Δ% 1.661; 95% CI 0.8, 2.5; *p* = 0.0298), and negatively with hsa-miR-520b-3p (Δ% −3.776; 95% CI −5.9, −1.6; *p* = 0.0919) and hsa-miR-638 (Δ% −4.530; 95% CI −7.1, −1.9; *p* = 0.0919) (Supplementary Table 2).

**Figure 4:**
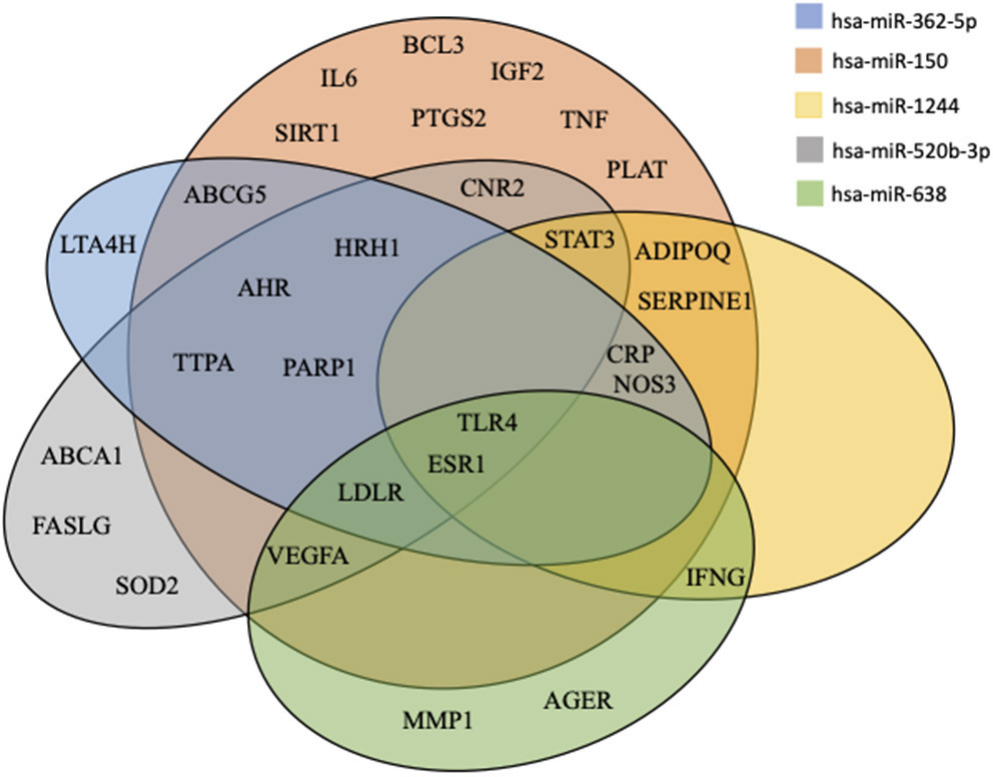
Venn diagram of miRNAs targets. All predicted genes for the differentially expressed miRNAs were filtered according to their relation with atherosclerosis and were selected for checking gene targets overlap. Results show the miRNAs target common genes.

For each of the five miRNAs, we considered genes predicted by at least two of the four evaluated algorithms to be *bona fide* target genes. The number of predicted target genes were 3,599 (for hsa-miR-362-5p), 7,032 (for hsa-miR-150), 2,812 (for hsa-miR-1244), 4,539 (for hsa-miR-520b-3p), and 1,882 (for hsa-miR-638). To elucidate the mechanisms through which the five EV-derived-miRNAs could impact on atherosclerosis, a miRNA-target interaction analysis was performed and these genes were compared to those found in the atherosclerosis gene network built using the disgenet2r package of R software. Fifty-nine genes were associated with atherosclerosis (Supplementary Figure 2). Among them, 29 atherosclerosis-related genes were the predicted targets of at least one of the five EV-derived-miRNAs (Supplementary Table 2). For the genes targeted by these miRNAs, we draw a Venn diagram showing common targets (Figure 4). Two genes were in common between all the 5 miRNAs [toll-like receptor 4 (TLR4) and estrogen receptor 1 (ERS1)], whereas the LDLR was the target of 4.

### Association Between the Four miRNAs Targeting LDLR *in silico*, and LDLR mRNA

As a proof-of-concept, we evaluated the mRNA expression of LDLR extracted from circulating leukocytes and we associated them with the levels of hsa-miR-362-5p, hsa-miR-150, hsa-miR-520b-3p, and hsa-miR-638, as these four miRNAs were found to target LDLR *in silico*. The sum of the four miRNAs was negatively associated to LDLR gene expression (β = −0.041; 95% CI −0.078, −0.004; *p* = 0.0296). The effect seems exclusively driven by has-miR-150 (Table 3), which was negatively associated with the gene expression of LDLR in a multivariable model considering all the four miRNAs (β_miR−150_ = −0.050; 95% CI −0.092, −0.008; *p* = 0.0193). Finally, considering that we were not able to validate these findings in atherosclerotic cardiovascular disease patients, we further subdivided our cohort according to the LDL-C risk threshold of 116 mg/dL (23). A positive association was found between PCSK9 and LDL-C (β = 0.41; SE = 0.10, *p* < 0.0001), with higher levels of PCSK9 in the group with LDL-C > 116 mg/dL (Supplementary Table 3). According to this stratification, the negative association between hsa-miR-150 and LDLR was present only in the group with LDL-c > 116 mg/dL and higher levels of PCSK9 (β_miR−150_ = −0.065; 95% CI −0.118, −0-011, *p* = 0.0182) (Supplementary Table 4).

**Table 3:** Association between miRNA levels (RQ) and LDL receptor (RQ) with multivariable linear regression models.

	**β**	**95% CI**	**P-value**
hsa-miR-362-5p + has-miR-150 + has-miR-520b-3p + hsa-miR-638	−0.041	−0.078; −0.004	0.0296
Multivariable model:			
hsa-miR-362-5p	0.021	–0.022; 0.063	0.3444
has-miR-150	−0.050	−0.092; −0.008	0.0193
has-miR-520b-3p	–0.016	–0.053; 0.021	0.3924
hsa-miR-638	–0.005	–0.042; 0.032	0.8014

*All linear regression models were adjusted for age, gender, BMI*.

*β regression coefficients were reported for one SD increment (SD_sum_of_four = 2425.44; SD_ hsa-miR-362-5p = 1.96; SD_ hsa-miR-150 = 2424.43; SD_ hsa-miR-638 = 6.93; SD_ hsa-miR-520b-3p = 10.55). BMI, body mass index*.

## Discussion

Considering that EVs are key players in the initiation of atherosclerosis and lesion progression (10), the results of the present study portend to a novel role for PCSK9 in regulating the intercellular communication mediated by these lipid bilayer membrane vesicles. In subjects with obesity, we found that PCSK9 may impact on the release of EVs-derived from atherosclerotic components (i.e., platelets, endothelium, monocytes/macrophages, and neutrophils) as well as on EVs-derived miRNA linked to atherosclerosis (hsa-miR-362, hsa-miR-150, hsa-miR-1244, hsa-miR-520b-3p, and hsa-miR-638) and their related targeted genes (e.g., LDLR, TLR4, and ESR1). Overall our data have to be interpreted in the context of the lack of clear mechanisms linking obesity and atherosclerosis. Considering that (i) in subjects with obesity, the number, and content of circulating plasma EVs are altered (24, 25), (ii) the range of cardiovascular pathologies in which EV are suspected to play a role is very wide, and (iii) EVs play pro- and anti-atherothrombotic effects (26), the identification of established atherosclerotic players able to regulate the biogenesis of EVs involved in atherosclerosis could be of high therapeutic interest. Indeed, although pharmacological reduction of PCSK9, through approved monoclonal antibodies, leads to 15% reduction in cardiovascular events, data on EVs changes in these patients are missing.

Atherosclerosis is a progressive, chronic inflammatory disease affecting large blood vessels by triggering the build-up of plaque within the vessel wall. The hypothesis that PCSK9 can be directly linked to atherogenesis is supported by observations that PCSK9 is expressed in vascular smooth muscle cells as well as in human atherosclerotic plaques (27). Further, the absence of PCSK9 is associated with a reduced neointimal formation, supporting the stimulatory effect of PCSK9 on intimal thickening (28). Vascular endothelial cells express and secrete functional PCSK9 under the control of shear stress which enhances PCSK9 expression in concert with reactive oxygen species (ROS). PCSK9 expression is also enhanced in macrophages by activation of the NLRP3 inflammasome and IL-1β (29). In human primary macrophages, exposure to human recombinant PCSK9 upregulated pro-inflammatory cytokines (30). PCSK9 enhances platelet responsiveness and arterial thrombosis (31) and it is considered as a danger-associated molecular pattern, similar to oxLDL, advanced glycated -proteins, S100A, and cell-derived microvesicles (32). Finally, PCSK9 positively correlates with white blood cells (WBC) and in particular with neutrophils (33), markers of cardiovascular disease (34).

In this framework, although no causality can be inferred by our findings, it becomes interesting to hypothesize an effect of PCSK9 on the release of EVs which are vehicles of bioactive molecules that influence various processes in atherosclerosis, namely, initiation, lesion progression, and hemostasis regulation (4). Although it is generally recognized that cardiovascular diseases are associated with a raise in EVs of a certain phenotype, e.g., of platelet origin (35), it has to be considered that our data were extrapolated from a cohort without overt cardiovascular diseases, at the time of recruitment (17). Thus, it is important to discuss the results we obtained in this light.

Platelet derived-EVs can act as a paracrine messenger that intensifies inflammation during the atherogenic process by stimulating vascular and inflammatory cells (10). Platelet-derived EVs support monocyte recruitment in large and small blood vessels, through a progressive transfer of the platelet adhesion receptor GPIbα to monocytes (36). Thus, since PCSK9 can influence platelet reactivity (32) and monocytes/macrophages cytokine release, it cannot be denied that it could regulate enriched CD61^+^ EVs according to the number of platelet count, i.e., when an individual has a low amount of platelet count there is a physiological increment in the release of EVs, whilst in the presence of a higher platelet count, there is a decrement in the release. Similar hypotheses can be drawn in the case of CD14^+^ and CD105^+^ enriched EVs. Considering that PCSK9 regulates both the release of cytokine from macrophages and ROS from endothelial cells, it was expected that its impact was mediated by inflammation. Indeed, macrophages are a major site of IL-8 production in atherosclerotic plaques (37), with IL-8 levels being positively associated with an increased risk of cardiovascular disease in apparently healthy individuals (38). Moreover, EVs can induce the release of IL-8 from endothelial cells and leukocytes, favoring the adhesion of monocytes (39). No further interactions have been found with other inflammatory markers, e.g., CRP.

Another important role might be exerted by EVs-derived miRNAs, as they have the potential to regulate the expression of genes at the transcriptional levels in recipient cells which are far from the source of EVs. For example, EVs released from endothelial cells might potentially transfer miRNAs to smooth muscle cells to restore communication (40). miRNA form a complex network of genes that control virtually every biological process (41), as well as progression and regression of atherosclerosis (41). The miRNAs we found to be associated with PCSK9 have been all previously reported to play a role in atherosclerosis. hsa-miR-150 enhances inflammatory responses by upregulating endothelial cell proliferation and migration, as well as intravascular environmental homeostasis. Therefore, it has been identified as a promising target for the management of atherosclerosis (42). On the contrary, hsa-miR-362 inhibits the proliferation and migration of vascular smooth muscle cells in atherosclerosis (43) supporting the ambivalent nature of EVs in this context. miR-520b-3p was shown to suppress endothelial inflammation and block the cross-talk between monocytes and endothelial cells by down-regulating NF-κB p65-ICAM1/VCAM1 axis (44). Thus, hsa-miR-520b-3p was downregulated in our data, suggesting a possible proinflammatory effect. hsa-miR-638 is a key molecule in regulating human vascular smooth muscle cell proliferation and migration (45). Interestingly, taking the network of target genes impacted by these miRNAs as a whole, it is suggestive to note that, besides LDLR, the majority of targets are related to inflammatory pathways (e.g., TLR4, STAT3, CRP, IL6, IFN-γ, TNF-α, etc.) pointing out that the role of PCSK9 on atherosclerosis has to be thought also in the context of an inflammatory autocrine/paracrine loop (46–49). As a proof-of-concept, the sum of the four miRNAs was negatively associated with gene expression of LDLR in leukocytes. Finally, relative to the regulation of ESR1 (ERα), it can control atherosclerotic calcification and smooth muscle cell osteogenic differentiation (50), mediates susceptibility to early atherosclerosis in male mice (51), and is directly involved in the regulation of cholesterol metabolism in macrophages (52).

These results should be interpreted within the context of potential limitations, e.g., this is an associative study which does not allow to infer any causality. Secondly, the isolation method involving ultracentrifugation did not allow to distinguish between microvesicles and exosomes. Whereas microvesicles are involved in enhancing blood clotting, exosomes suppress platelet aggregation and occlusive thrombi by inhibiting CD36 (53). Third, EV total count might have been influenced by HDL and LDL, which share the same density and size as EVs. However, as PCSK9 is expected to be associated with LDL, and the associations we observed between PCSK9 and EVs were negative, it is unplausible that the observed results are due to this contamination. On the contrary, as HDL transport several miRNAs, it is possible that the observed changes are also related to these circulating components. Fourth, there is a lack of data on EVs released from smooth muscle cells. Fifth, although the lack of follow-up did not allow to test the effectiveness of EVs as a biomarker of atherosclerosis, a follow-up recording of cardiovascular outcomes is on going.

## Conclusions

In the context of the atherosclerotic process, the present findings show a potential role of PCSK9 on the complex intercellular communication routes involving a network of cells and their EVs-derived miRNAs.

## Data Availability Statement

The original contributions presented in the study are included in the article/Supplementary Material, further inquiries can be directed to the corresponding authors.

## Ethics Statement

The studies involving human participants were reviewed and approved by Fondazione IRCCS Cà Granda Ospedale Maggiore Policlinico (approval number 1425). The patients/participants provided their written informed consent to participate in this study.

## Author Contributions

CM and MG performed the experiments and drafted the manuscript. CF performed the statistical analysis. LD, LC, and MH characterized the EVs. GS performed the analysis of miRNAs. LV was responsible for the recruitment of SPHERE cohort. AC, MB, and ACP critically reviewed the manuscript. VB and MR conceived the study and wrote the manuscript. All authors contributed to the article and approved the submitted version.

## Funding

This work was supported by Fondazione Cariplo (2018-0511 to MR); European Research Council (ERC-2011-StG 282,413 to VB).

## Conflict of Interest

The authors declare that the research was conducted in the absence of any commercial or financial relationships that could be construed as a potential conflict of interest. The reviewer PM declared a shared affiliation, with one of the authors LV to the handling editor at the time of the review.

## Publisher's Note

All claims expressed in this article are solely those of the authors and do not necessarily represent those of their affiliated organizations, or those of the publisher, the editors and the reviewers. Any product that may be evaluated in this article, or claim that may be made by its manufacturer, is not guaranteed or endorsed by the publisher.
