# Supplementary material for: Associations Among PCSK9 Levels, Atherosclerosis-Derived Extracellular Vesicles, and Their miRNA Content in Adults With Obesity
Source: Front Cardiovasc Med. 2022 Jan 7;8:785250. doi: 10.3389/fcvm.2021.785250 (PMC8782054; doi:10.3389/fcvm.2021.785250)
Supplement: Supplementary file 1 [file Data_Sheet_1.docx]

**SUPPLEMENTAL MATERIALS**

**Title:** Associations among PCSK9 levels, atherosclerosis-derived extracellular vesicles, and their miRNA content in adults with obesity

Chiara Macchi^#1^, Maria Francesca Greco^#1^, Chiara Favero^2^, Laura Dioni^2^, Laura Cantone^2^, Mirjam Hoxha ^2^, Luisella Vigna^3^, Giulia Solazzo^2^, Alberto Corsini^1,4^, Maciej Banach^5,6^, Angela C Pesatori^2^, Valentina Bollati^§2^ and Massimiliano Ruscica^§1^

^1^Department of Pharmacological and Biomolecular Sciences, Università degli Studi di Milano, Italy^; 2^EPIGET Department of Clinical Sciences and Community Health, Università degli Studi di Milano, Milan, Italy; ^3^ Occupational Medicine Unit, Fondazione Cà Granda, IRCCS Ospedale Maggiore Policlinico, Milan, Italy;^4^ IRCCS, Multimedica, Sesto San Giovanni, Milan; ^5^Department of Preventive Cardiology and Lipidology, Medical University of Lodz, 93-338 Lodz, Poland; ^6^Cardiovascular Research Centre, University of Zielona Gora, 65-046 Zielona Gora, Poland.

**Supplemental Table 1**

**Supplemental Table 2**

**Supplemental Table 3**

**Supplemental Table 4**

**Supplemental Figure 1**

**Supplemental Figure 2**

**Supplemental legend Figure 1**

**Supplemental Table 1.** Quantification of EV fraction and EV subtypes

|  | **EV characteristics** | **Median [Q1-Q3]** | **Range** |
| --- | --- | --- | --- |
| **EV count (10^6^/ml plasma) ^a^** | **EV count (n)** | 1912 [1352 - 2513] | 182 - 70945 |
|  | **Average size (nm)** | 212 [192 - 230] | 95 - 317 |
|  | **Mode size (nm)** | 155 [137 - 170] | 66 - 233 |
|  |  |  |  |
| **EV subtypes (10^3^/ml plasma) ^b^** | **EV CD66^+^ (neutrophils)** | 15.3 [8.7 - 25.3] | 0.33 - 224 |
|  | **EV EpCAM^+^ (epithelium)** | 12 [7 - 21] | 0.33 - 147 |
|  | **EV CD105^+^ (endothelium)** | 9.7 [6 - 15] | 0.33 - 49 |
|  | **EV CD14^+^ (macrophages/monocytes)** | 18 [9.7 - 33] | 0.67 - 821 |
|  | **EV CD61^+^ (platelets)** | 91.3 [45 - 168] | 1.33 - 2351 |
|  |  |  |  |
| EV, Extracellular Vesicles; Q1-Q3, first quartile - third quartile.  ^a^ Count of EV obtained from Nanosight analysis.  ^b^ Count of EV subtypes obtained from flow cytometry. | | | |

**Supplemental Table 2.**  Association between PCSK9 levels and EV-miRNA levels measured by Open Array.

Δ% represents the percent variation ((1 – exp(β))*100) in EV-miRNA levels for 10 ng/mL increase in PCSK9 concentrations.
All linear regression models were adjusted for age, gender, BMI, smoking habit, statin use, PM_10_ and apparent temperature at the day before the blood draw.

| **Quant Studio Assay ID** | **Assay Name** | **miRBase ID (v21)** | **miRBase Alias** | **Δ%** | **95% CI** | | **P-value** | **FDR  P-value** |
| --- | --- | --- | --- | --- | --- | --- | --- | --- |
| 000186 | hsa-miR-150 | hsa-miR-150-5p, | hsa-miR-150(17), | 5.238 | 2.515 | 8.032 | **0.0001** | **0.0298** |
| 000268 | hsa-miR-362 | hsa-miR-362-5p, | hsa-miR-362(9.2) | 3.069 | 1.569 | 4.590 | **0.0001** | **0.0298** |
| 000377 | hsa-miR-1244 | hsa-miR-1244, | NA | 1.661 | 0.795 | 2.535 | **0.0002** | **0.0298** |
| 000379 | hsa-miR-520b | hsa-miR-520b, |  | -3.776 | -5.921 | -1.581 | **0.0008** | **0.0919** |
| 000382 | hsa-miR-638 | hsa-miR-638 | NA | -4.530 | -7.094 | -1.895 | **0.0009** | **0.0919** |
| 000387 | hsa-miR-31 | hsa-miR-31-5p | hsa-miR-31(17) | 4.040 | 1.460 | 6.687 | **0.0020** | **0.1325** |
| 000389 | hsa-miR-20a# | hsa-miR-20a-3p | hsa-miR-20a*(17) | 2.301 | 0.801 | 3.823 | **0.0026** | **0.1325** |
| 000390 | hsa-miR-539 | hsa-miR-539-5p, | hsa-miR-539(17), | 2.964 | 1.015 | 4.952 | **0.0028** | **0.1325** |
| 000391 | hsa-miR-1260 | hsa-miR-1260a, | hsa-miR-1260(17), | -3.662 | -5.992 | -1.275 | **0.0029** | **0.1325** |
| 000395 | hsa-miR-493 | hsa-miR-493-3p, | hsa-miR-493(17), | 1.254 | 0.426 | 2.090 | **0.0030** | **0.1325** |
| 000396 | hsa-miR-155 | hsa-miR-155-5p, | hsa-miR-155(17), | 5.324 | 1.744 | 9.030 | **0.0033** | **0.1325** |
| 000397 | hsa-miR-125b-1# | hsa-miR-125b-1-3p, | hsa-miR-125b-1*(17), | -1.891 | -3.138 | -0.628 | **0.0035** | **0.1325** |
| 000398 | hsa-miR-211 | hsa-miR-211-5p, | hsa-miR-211(17) | -3.140 | -5.202 | -1.032 | **0.0037** | **0.1325** |
| 000399 | hsa-miR-520c-3p | hsa-miR-520c-3p, | NA | -5.474 | -8.999 | -1.813 | **0.0037** | **0.1325** |
| 000400 | hsa-miR-1271 | hsa-miR-1271-5p, | hsa-miR-1271(17), | -1.728 | -2.880 | -0.563 | **0.0038** | **0.1325** |
| 000402 | hsa-miR-135b | hsa-miR-135b-5p, | hsa-miR-135b(17), | -1.792 | -2.992 | -0.576 | **0.0040** | **0.1325** |
| 000403 | hsa-miR-140 | hsa-miR-140-5p, |  | 2.308 | 0.708 | 3.933 | **0.0046** | **0.1432** |
| 000405 | hsa-miR-183# | hsa-miR-183-3p, | hsa-miR-183*(17), | -2.282 | -3.839 | -0.699 | **0.0049** | **0.1443** |
| 000407 | hsa-miR-302c# | hsa-miR-302c-5p, | hsa-miR-302c*(17) | -3.309 | -5.574 | -0.990 | **0.0055** | **0.1513** |
| 000408 | hsa-miR-125a-3p | hsa-miR-125a-3p, | NA | -1.534 | -2.650 | -0.406 | **0.0079** | **0.2078** |
| 000409 | hsa-miR-628-5p | hsa-miR-628-5p, | NA | 7.169 | 1.521 | 13.131 | **0.0123** | 0.3079 |
| 000411 | hsa-miR-340# | hsa-miR-340-3p, | hsa-miR-340*(17) | 2.037 | 0.395 | 3.707 | **0.0149** | 0.3551 |
| 000413 | hsa-miR-29c | hsa-miR-29c-3p, | hsa-miR-29c(17), | 1.873 | 0.355 | 3.413 | **0.0155** | 0.3551 |
| 000416 | hsa-miR-190 | hsa-miR-190a-5p, | hsa-miR-190(17),hsa-miR-190a(19), | 1.868 | 0.330 | 3.429 | **0.0172** | 0.3739 |
| 000417 | hsa-miR-505 | hsa-miR-505-3p, | hsa-miR-505(17), | -1.970 | -3.569 | -0.344 | **0.0178** | 0.3739 |
| 000419 | hsa-miR-326 | hsa-miR-326, | NA | -2.017 | -3.674 | -0.331 | **0.0193** | 0.3739 |
| 000420 | hsa-miR-126 | hsa-miR-126-3p, | hsa-miR-126(17), | 2.562 | 0.392 | 4.778 | **0.0205** | 0.3739 |
| 000422 | hsa-miR-103 | hsa-miR-103a-3p, | hsa-miR-103(16),hsa-miR-103a(17), | 2.113 | 0.314 | 3.945 | **0.0212** | 0.3739 |
| 000426 | hsa-miR-29a | hsa-miR-29a-3p, | hsa-miR-29a(17), | 2.418 | 0.352 | 4.527 | **0.0217** | 0.3739 |
| 000428 | hsa-miR-590-3P | hsa-miR-590-3p, | NA | 2.042 | 0.276 | 3.840 | **0.0233** | 0.3739 |
| 000431 | hsa-miR-545# | hsa-miR-545-5p | hsa-miR-545*(17) | -1.077 | -1.998 | -0.147 | **0.0234** | 0.3739 |
| 000433 | hsa-miR-659 | hsa-miR-659-3p | hsa-miR-659(17) | -1.556 | -2.884 | -0.210 | **0.0237** | 0.3739 |
| 000435 | hsa-miR-30c | hsa-miR-30c-5p, | hsa-miR-30c(17), | 3.903 | 0.490 | 7.433 | **0.0247** | 0.3739 |
| 000436 | hsa-miR-142-3p | hsa-miR-142-3p, |  | 1.707 | 0.203 | 3.234 | **0.0260** | 0.3739 |
| 000437 | hsa-miR-603 | hsa-miR-603, | NA | -1.513 | -2.832 | -0.175 | **0.0268** | 0.3739 |
| 000439 | hsa-miR-30b | hsa-miR-30b-5p, | hsa-miR-30b(17), | 3.060 | 0.335 | 5.859 | **0.0275** | 0.3739 |
| 000442 | hsa-miR-411 | hsa-miR-411-5p, | hsa-miR-411(17), | 2.469 | 0.252 | 4.735 | **0.0289** | 0.3739 |
| 000443 | hsa-miR-1303 | hsa-miR-1303, | NA | -0.910 | -1.719 | -0.094 | **0.0290** | 0.3739 |
| 000449 | hsa-miR-16 | hsa-miR-16-5p, | hsa-miR-16(17), | 1.582 | 0.156 | 3.028 | **0.0296** | 0.3739 |
| 000451 | hsa-miR-191 | hsa-miR-191-5p, | hsa-miR-191(17), | 1.469 | 0.140 | 2.816 | **0.0302** | 0.3739 |
| 000452 | hsa-miR-338-5P |  |  | -1.566 | -2.963 | -0.149 | **0.0305** | 0.3739 |
| 000454 | hsa-miR-374 | hsa-miR-374a-5p, | hsa-miR-374(9.2),hsa-miR-374a(17), | 1.644 | 0.141 | 3.169 | **0.0319** | 0.3739 |
| 000456 | hsa-miR-146b | hsa-miR-146b-5p, | hsa-miR-146b(9.2), | 1.618 | 0.134 | 3.124 | **0.0326** | 0.3739 |
| 000457 | hsa-miR-369-3p | hsa-miR-369-3p, | hsa-miR-369(6) | 1.879 | 0.151 | 3.637 | **0.0330** | 0.3739 |
| 000460 | hsa-miR-433 | hsa-miR-433-3p, | hsa-miR-433(19), | 1.362 | 0.108 | 2.631 | **0.0332** | 0.3739 |
| 000463 | hsa-miR-22 | hsa-miR-22-3p, | hsa-miR-22(17), | -3.124 | -5.914 | -0.252 | **0.0333** | 0.3739 |
| 000464 | hsa-miR-139-5p |  | hsa-miR-139-5p(18) | 1.652 | 0.128 | 3.199 | **0.0335** | 0.3739 |
| 000468 | hsa-miR-551b# | hsa-miR-551b-5p | hsa-miR-551b*(17) | -1.667 | -3.187 | -0.124 | **0.0343** | 0.3739 |
| 000469 | hsa-miR-204 | hsa-miR-204-5p, | hsa-miR-204(17), | 2.354 | 0.167 | 4.588 | **0.0348** | 0.3739 |
| 000470 | hsa-miR-649 | hsa-miR-649, | NA | -1.255 | -2.425 | -0.072 | **0.0377** | 0.3899 |
| 000471 | hsa-miR-370 | hsa-miR-370-3p, | hsa-miR-370(19), | 3.484 | 0.195 | 6.882 | **0.0377** | 0.3899 |
| 000473 | hsa-miR-553 | hsa-miR-553, | NA | -1.248 | -2.428 | -0.054 | **0.0406** | 0.391 |
| 000475 | hsa-miR-548P | hsa-miR-548p, | NA | -1.465 | -2.850 | -0.060 | **0.0411** | 0.391 |
| 000477 | hsa-miR-597 | hsa-miR-597-5p, | hsa-miR-597(19) | 1.786 | 0.069 | 3.532 | **0.0414** | 0.391 |
| 000478 | hsa-miR-98 | hsa-miR-98-5p, | hsa-miR-98(18), | 3.699 | 0.140 | 7.383 | **0.0415** | 0.391 |
| 000480 | hsa-miR-30b# | hsa-miR-30b-3p, | hsa-miR-30b*(17) | -1.205 | -2.356 | -0.041 | **0.0426** | 0.391 |
| 000482 | hsa-miR-151-5P | hsa-miR-151a-5p, | hsa-miR-151-5p(17), | 1.736 | 0.057 | 3.444 | **0.0427** | 0.391 |
| 000485 | hsa-miR-190b | hsa-miR-190b, | mmu-miR-190b(17) | -1.452 | -2.838 | -0.046 | **0.0430** | 0.391 |
| 000489 | hsa-miR-200c | hsa-miR-200c-3p, | hsa-miR-200c(17), | -1.839 | -3.596 | -0.051 | **0.0439** | 0.3915 |
| 000491 | hsa-miR-378 |  | hsa-miR-378(17),hsa-miR-378a-3p(19), | -1.809 | -3.542 | -0.044 | **0.0446** | 0.3915 |
| 000493 | hsa-miR-708 | hsa-miR-708-5p, | hsa-miR-708(17), | 1.616 | 0.033 | 3.225 | **0.0454** | 0.3919 |
| 000494 | hsa-miR-107 | hsa-miR-107, |  | 2.144 | 0.007 | 4.326 | **0.0493** | 0.4109 |
| 000497 | hsa-miR-604 | hsa-miR-604 | NA | -0.827 | -1.646 | -0.002 | **0.0495** | 0.4109 |
| 000498 | hsa-miR-651 | hsa-miR-651-5p | hsa-miR-651(19) | -1.002 | -1.993 | 0.000 | **0.0499** | 0.4109 |
| 000500 | hsa-miR-1252 | hsa-miR-1252-5p | hsa-miR-1252(19) | -1.170 | -2.339 | 0.013 | 0.0524 | 0.4183 |
| 000502 | hsa-miR-622 | hsa-miR-622, | NA | -0.951 | -1.907 | 0.016 | 0.0538 | 0.4183 |
| 000507 | hsa-miR-192# | hsa-miR-192-3p, | hsa-miR-192*(17), | -0.955 | -1.917 | 0.016 | 0.0540 | 0.4183 |
| 000508 | hsa-miR-18a# | hsa-miR-18a-3p | hsa-miR-18a*(17) | -0.952 | -1.911 | 0.016 | 0.0540 | 0.4183 |
| 000509 | hsa-miR-93# | hsa-miR-93-3p, | hsa-miR-93*(17), | 1.428 | -0.030 | 2.907 | 0.0549 | 0.4191 |
| 000510 | hsa-miR-186 | hsa-miR-186-5p, | hsa-miR-186(17), | 1.636 | -0.040 | 3.341 | 0.0557 | 0.4191 |
| 000511 | hsa-let-7d | hsa-let-7d-5p, | hsa-let-7d(17), | 2.044 | -0.058 | 4.191 | 0.0568 | 0.4191 |
| 000512 | hsa-miR-1228# | hsa-miR-1228-5p | hsa-miR-1228*(17) | -0.841 | -1.706 | 0.032 | 0.0590 | 0.4191 |
| 000514 | hsa-miR-222# | hsa-miR-222-5p | hsa-miR-222*(17) | -0.812 | -1.650 | 0.032 | 0.0594 | 0.4191 |
| 000515 | hsa-miR-1292 | hsa-miR-1292-5p, | hsa-miR-1292(18) | -0.835 | -1.695 | 0.033 | 0.0594 | 0.4191 |
| 000516 | hsa-miR-550 | hsa-miR-550a-5p, | hsa-miR-550(15),hsa-miR-550a(17), | -1.255 | -2.550 | 0.057 | 0.0608 | 0.4191 |
| 000518 | hsa-miR-153 | hsa-miR-153-3p, | hsa-miR-153(19), | -0.769 | -1.570 | 0.038 | 0.0619 | 0.4191 |
| 000521 | hsa-miR-570 | hsa-miR-570-3p | hsa-miR-570(17) | -0.769 | -1.570 | 0.038 | 0.0619 | 0.4191 |
| 000522 | hsa-let-7f-1# | hsa-let-7f-1-3p, | hsa-let-7f-1*(17), | -0.801 | -1.636 | 0.041 | 0.0620 | 0.4191 |
| 000524 | hsa-miR-223# | hsa-miR-223-5p | hsa-miR-223*(17) | 1.439 | -0.082 | 2.983 | 0.0637 | 0.4249 |
| 000527 | hsa-miR-1291 | hsa-miR-1291, | NA | -1.458 | -2.986 | 0.095 | 0.0656 | 0.4318 |
| 000528 | hsa-miR-1227 | hsa-miR-1227-3p | hsa-miR-1227(18) | -0.864 | -1.779 | 0.059 | 0.0664 | 0.4321 |
| 000529 | hsa-miR-126# | hsa-miR-126-5p, | hsa-miR-126*(17), | 2.162 | -0.174 | 4.552 | 0.0699 | 0.433 |
| 000531 | hsa-miR-548c | hsa-miR-548c-3p, | hsa-miR-548c(9.2) | -1.278 | -2.642 | 0.105 | 0.0699 | 0.433 |
| 000533 | hsa-miR-181a | hsa-miR-181a-5p, | hsa-miR-181a(17), | 2.318 | -0.198 | 4.898 | 0.0712 | 0.433 |
| 000534 | hsa-miR-23a# | hsa-miR-23a-5p, | hsa-miR-23a*(17), | -0.746 | -1.552 | 0.066 | 0.0715 | 0.433 |
| 000535 | hsa-miR-508 | hsa-miR-508-3p | hsa-miR-508(9.2) | -0.890 | -1.851 | 0.080 | 0.0718 | 0.433 |
| 000539 | hsa-miR-572 | hsa-miR-572, | NA | -2.373 | -4.903 | 0.224 | 0.0729 | 0.433 |
| 000540 | hsa-miR-488 | hsa-miR-488-5p, | hsa-miR-488(9.2),hsa-miR-488*(17), | -0.738 | -1.542 | 0.073 | 0.0744 | 0.433 |
| 000542 | hsa-miR-585 | hsa-miR-585-3p | hsa-miR-585(19) | -1.054 | -2.200 | 0.106 | 0.0747 | 0.433 |
| 000543 | hsa-miR-142-5p | hsa-miR-142-5p, |  | 1.528 | -0.161 | 3.246 | 0.0763 | 0.433 |
| 000544 | hsa-miR-146a | hsa-miR-146a-5p, | hsa-miR-146(6),hsa-miR-146a(17), | 1.896 | -0.200 | 4.036 | 0.0764 | 0.433 |
| 000545 | hsa-miR-502 | hsa-miR-502-5p, | hsa-miR-502(9.2) | 1.382 | -0.146 | 2.932 | 0.0764 | 0.433 |
| 000546 | hsa-miR-138 | hsa-miR-138-5p, | hsa-miR-138(17), | 1.638 | -0.173 | 3.481 | 0.0764 | 0.433 |
| 000554 | hsa-miR-146b-3p | hsa-miR-146b-3p, | NA | 1.216 | -0.136 | 2.585 | 0.0780 | 0.433 |
| 000555 | hsa-miR-203 | hsa-miR-203a-3p, | hsa-miR-203(18),hsa-miR-203a(20), | 1.680 | -0.198 | 3.593 | 0.0797 | 0.433 |
| 000557 | hsa-miR-655 | hsa-miR-655-3p, | hsa-miR-655(19) | -0.940 | -1.984 | 0.114 | 0.0802 | 0.433 |
| 000560 | hsa-miR-516-3p |  |  | -0.743 | -1.572 | 0.092 | 0.0811 | 0.433 |
| 000561 | hsa-miR-379 | hsa-miR-379-5p, | hsa-miR-379(17), | 1.743 | -0.221 | 3.746 | 0.0822 | 0.433 |
| 000563 | hsa-miR-623 | hsa-miR-623 | NA | 2.287 | -0.292 | 4.932 | 0.0825 | 0.433 |
| 000564 | hsa-miR-224 | hsa-miR-224-5p | hsa-miR-224(17) | 1.745 | -0.223 | 3.753 | 0.0825 | 0.433 |
| 000565 | hsa-miR-29b | hsa-miR-29b-3p, | hsa-miR-29b(17), | 1.523 | -0.198 | 3.273 | 0.0830 | 0.433 |
| 000566 | hsa-miR-99a# | hsa-miR-99a-3p | hsa-miR-99a*(17) | -0.712 | -1.514 | 0.096 | 0.0840 | 0.434 |
| 000567 | hsa-miR-542-5p | hsa-miR-542-5p, | NA | -0.706 | -1.507 | 0.102 | 0.0865 | 0.4358 |
| 000569 | hsa-miR-15b | hsa-miR-15b-5p, | hsa-miR-15b(17), | 1.727 | -0.248 | 3.741 | 0.0868 | 0.4358 |
| 000570 | hsa-miR-340 | hsa-miR-340-5p, | hsa-miR-340(17), | 1.571 | -0.230 | 3.405 | 0.0875 | 0.4358 |
| 000571 | hsa-miR-409-3p | hsa-miR-409-3p, | NA | 2.249 | -0.329 | 4.894 | 0.0876 | 0.4358 |
| 000572 | hsa-miR-519b-3p | hsa-miR-519b-3p, | NA | -0.693 | -1.503 | 0.123 | 0.0955 | 0.4705 |
| 000573 | hsa-miR-339-5p | hsa-miR-339-5p, | NA | 3.187 | -0.581 | 7.098 | 0.0982 | 0.4793 |
| 000574 | hsa-miR-181c# | hsa-miR-181c-3p | hsa-miR-181c*(17) | -0.825 | -1.797 | 0.157 | 0.0994 | 0.4804 |
| 000577 | hsa-miR-1300 | NA | hsa-miR-1300(13), | 0.539 | -0.104 | 1.187 | 0.1005 | 0.481 |
| 000580 | hsa-miR-127 | hsa-miR-127-3p, | hsa-miR-127(9.2), | 1.685 | -0.333 | 3.743 | 0.1021 | 0.481 |
| 000583 | hsa-miR-338-3p | hsa-miR-338-3p, | tgu-miR-338(15) | -0.698 | -1.528 | 0.140 | 0.1022 | 0.481 |
| 000587 | hsa-miR-1243 | hsa-miR-1243 | NA | 8.385 | -1.741 | 19.555 | 0.1075 | 0.5005 |
| 000592 | hsa-miR-181a-2# | hsa-miR-181a-2-3p | hsa-miR-181a-2*(17) | 1.302 | -0.287 | 2.917 | 0.1085 | 0.5005 |
| 000600 | hsa-miR-34b | hsa-miR-34b-3p, | hsa-miR-34b(17) | -0.505 | -1.120 | 0.114 | 0.1093 | 0.5005 |
| 000602 | hsa-miR-33a# | hsa-miR-33a-3p, | hsa-miR-33a*(17), | -0.701 | -1.557 | 0.163 | 0.1113 | 0.5005 |
| 000604 | hsa-miR-23a | hsa-miR-23a-3p, | hsa-miR-23a(17), | -2.213 | -4.882 | 0.530 | 0.1127 | 0.5005 |
| 001014 | hsa-miR-486-3p | hsa-miR-486-3p, |  | -0.518 | -1.154 | 0.123 | 0.1130 | 0.5005 |
| 001020 | hsa-miR-517a | hsa-miR-517a-3p,hsa-miR-517b-3p, | hsa-miR-517a(17) | -1.143 | -2.539 | 0.273 | 0.1130 | 0.5005 |
| 001024 | hsa-miR-28 | hsa-miR-28-5p, | hsa-miR-28(9.2), | 1.413 | -0.337 | 3.195 | 0.1140 | 0.5005 |
| 001026 | hsa-miR-193b | hsa-miR-193b-3p, | hsa-miR-193b(17), | 1.320 | -0.324 | 2.990 | 0.1159 | 0.5047 |
| 001027 | hsa-miR-484 | hsa-miR-484, | NA | 2.258 | -0.577 | 5.174 | 0.1194 | 0.5126 |
| 001028 | hsa-miR-296 | hsa-miR-296-5p, | hsa-miR-296(9.2), | -2.190 | -4.884 | 0.580 | 0.1200 | 0.5126 |
| 001043 | hsa-miR-425# | hsa-miR-425-3p, | hsa-miR-425*(17) | 1.379 | -0.365 | 3.153 | 0.1217 | 0.5126 |
| 001046 | hsa-miR-758 | hsa-miR-758-3p, | hsa-miR-758(18), | 2.109 | -0.559 | 4.848 | 0.1221 | 0.5126 |
| 001048 | hsa-miR-1 | hsa-miR-1-3p, | hsa-miR-1(20), | -1.058 | -2.385 | 0.288 | 0.1226 | 0.5126 |
| 001052 | hsa-miR-503 | hsa-miR-503-5p, | hsa-miR-503(18), | 1.071 | -0.301 | 2.462 | 0.1264 | 0.5213 |
| 001090 | hsa-miR-454 | hsa-miR-454-3p, | hsa-miR-454(17), | 1.644 | -0.462 | 3.794 | 0.1266 | 0.5213 |
| 001097 | hsa-let-7c | hsa-let-7c-5p, | hsa-let-7c(19), | 1.242 | -0.377 | 2.888 | 0.1333 | 0.5444 |
| 001101 | hsa-miR-30d | hsa-miR-30d-5p, | hsa-miR-30d(17), | 1.189 | -0.385 | 2.788 | 0.1393 | 0.5631 |
| 001102 | hsa-let-7g | hsa-let-7g-5p, | hsa-let-7g(17), | 1.151 | -0.378 | 2.704 | 0.1406 | 0.5631 |
| 001106 | hsa-miR-31# | hsa-miR-31-3p | hsa-miR-31*(17) | 0.998 | -0.331 | 2.346 | 0.1415 | 0.5631 |
| 001109 | hsa-miR-20b | hsa-miR-20b-5p, | hsa-miR-20b(17), | 1.431 | -0.479 | 3.378 | 0.1427 | 0.5631 |
| 001111 | hsa-miR-628-3p | hsa-miR-628-3p, | NA | 0.766 | -0.264 | 1.807 | 0.1452 | 0.5631 |
| 001113 | hsa-miR-125a-5p | hsa-miR-125a-5p, | NA | -1.627 | -3.778 | 0.572 | 0.1455 | 0.5631 |
| 001116 | hsa-miR-26b | hsa-miR-26b-5p, | hsa-miR-26b(17), | 0.967 | -0.338 | 2.289 | 0.1467 | 0.5631 |
| 001119 | hsa-miR-376c | hsa-miR-376c-3p, | hsa-miR-376c(18), | 1.559 | -0.546 | 3.707 | 0.1475 | 0.5631 |
| 001120 | hsa-miR-381 | hsa-miR-381-3p, | hsa-miR-381(18), | 2.199 | -0.786 | 5.273 | 0.1501 | 0.5631 |
| 001129 | hsa-miR-335 | hsa-miR-335-5p, | hsa-miR-335(17), | -1.567 | -3.674 | 0.586 | 0.1523 | 0.5631 |
| 001138 | hsa-miR-320 | hsa-miR-320a, | hsa-miR-320(10.1), | 0.970 | -0.370 | 2.328 | 0.1566 | 0.5631 |
| 001141 | hsa-miR-26a-2# | hsa-miR-26a-2-3p, | hsa-miR-26a-2*(17) | -0.885 | -2.098 | 0.343 | 0.1567 | 0.5631 |
| 001149 | hsa-miR-1274B | NA | hsa-miR-1274b(16), | 1.069 | -0.409 | 2.569 | 0.1567 | 0.5631 |
| 001150 | hsa-miR-548L | hsa-miR-548l, | NA | -0.913 | -2.164 | 0.355 | 0.1573 | 0.5631 |
| 001153 | hsa-miR-518f | hsa-miR-518f-3p, | hsa-miR-518f(17) | -1.352 | -3.199 | 0.530 | 0.1576 | 0.5631 |
| 001156 | hsa-miR-586 | hsa-miR-586, | NA | -0.924 | -2.194 | 0.362 | 0.1580 | 0.5631 |
| 001159 | hsa-miR-28-3p | hsa-miR-28-3p, | rno-miR-28*(18) | 1.647 | -0.634 | 3.980 | 0.1581 | 0.5631 |
| 001173 | hsa-miR-770-5p | hsa-miR-770-5p, | NA | -0.909 | -2.160 | 0.357 | 0.1583 | 0.5631 |
| 001174 | hsa-miR-27b# | hsa-miR-27b-5p, | hsa-miR-27b*(17), | 0.646 | -0.251 | 1.552 | 0.1585 | 0.5631 |
| 001178 | hsa-miR-182 | hsa-miR-182-5p, | hsa-miR-182(17) | -1.046 | -2.489 | 0.418 | 0.1602 | 0.5631 |
| 001182 | hsa-miR-29b-2# | hsa-miR-29b-2-5p, | hsa-miR-29b-2*(17), | -0.827 | -1.970 | 0.330 | 0.1603 | 0.5631 |
| 001184 | hsa-miR-181c | hsa-miR-181c-5p, | hsa-miR-181c(17), | 1.250 | -0.522 | 3.053 | 0.1675 | 0.5639 |
| 001186 | hsa-miR-664 | hsa-miR-664a-3p, | hsa-miR-664(17),hsa-miR-664-3p(18), | -1.772 | -4.236 | 0.756 | 0.1676 | 0.5639 |
| 001187 | hsa-miR-302a | hsa-miR-302a-3p, | hsa-miR-302(3.1),hsa-miR-302a(17), | -1.360 | -3.267 | 0.585 | 0.1689 | 0.5639 |
| 001191 | hsa-miR-323-3p | hsa-miR-323a-3p, | hsa-miR-323-3p(17), | -1.090 | -2.628 | 0.472 | 0.1700 | 0.5639 |
| 001271 | hsa-miR-10a | hsa-miR-10a-5p, | hsa-miR-10a(17), | 1.135 | -0.490 | 2.787 | 0.1717 | 0.5639 |
| 001273 | hsa-miR-15a# | hsa-miR-15a-3p, | hsa-miR-15a*(17), | -0.858 | -2.076 | 0.376 | 0.1718 | 0.5639 |
| 001274 | hsa-miR-645 | hsa-miR-645, | NA | -1.133 | -2.739 | 0.498 | 0.1720 | 0.5639 |
| 001277 | hsa-miR-1274A | NA | hsa-miR-1274a(16) | 1.976 | -0.849 | 4.881 | 0.1720 | 0.5639 |
| 001278 | hsa-miR-136 | hsa-miR-136-5p, | hsa-miR-136(17), | 2.699 | -1.161 | 6.710 | 0.1728 | 0.5639 |
| 001279 | hsa-miR-1208 | hsa-miR-1208, | NA | -0.585 | -1.420 | 0.258 | 0.1731 | 0.5639 |
| 001280 | hsa-miR-548a | hsa-miR-548a-3p, | hsa-miR-548a(9.2) | -1.050 | -2.542 | 0.466 | 0.1734 | 0.5639 |
| 001284 | hsa-miR-133b | hsa-miR-133b, |  | -0.600 | -1.461 | 0.269 | 0.1752 | 0.5639 |
| 001285 | hsa-miR-135a | hsa-miR-135a-5p, | hsa-miR-135(3),hsa-miR-135a(17), | 1.170 | -0.519 | 2.887 | 0.1753 | 0.5639 |
| 001286 | hsa-miR-197 | hsa-miR-197-3p, | hsa-miR-197(17), | -3.325 | -7.944 | 1.526 | 0.1755 | 0.5639 |
| 001319 | hsa-miR-22# | hsa-miR-22-5p, | hsa-miR-22*(17), | 1.133 | -0.507 | 2.800 | 0.1766 | 0.5641 |
| 001338 | hsa-miR-523 | hsa-miR-523-3p, | hsa-miR-523(17) | -1.778 | -4.321 | 0.832 | 0.1797 | 0.5694 |
| 001510 | hsa-miR-635 | hsa-miR-635, | NA | -0.841 | -2.059 | 0.393 | 0.1804 | 0.5694 |
| 001514 | hsa-miR-630 | hsa-miR-630, | NA | -0.869 | -2.135 | 0.414 | 0.1831 | 0.57 |
| 001515 | hsa-miR-495 | hsa-miR-495-3p, | hsa-miR-495(18), | -1.820 | -4.444 | 0.878 | 0.1838 | 0.57 |
| 001516 | hsa-miR-518a-3p | hsa-miR-518a-3p, | NA | -0.855 | -2.103 | 0.409 | 0.1839 | 0.57 |
| 001518 | hsa-miR-1178 | hsa-miR-1178-3p, | hsa-miR-1178(18) | -0.695 | -1.714 | 0.335 | 0.1849 | 0.57 |
| 001521 | hsa-miR-373 | hsa-miR-373-3p, | hsa-miR-373(17) | -0.835 | -2.063 | 0.408 | 0.1865 | 0.5715 |
| 001528 | hsa-miR-34c | hsa-miR-34c-5p, | hsa-miR-34c(9.2), | -1.859 | -4.580 | 0.940 | 0.1906 | 0.577 |
| 001531 | hsa-miR-766 | hsa-miR-766-3p, | hsa-miR-766(17) | 0.997 | -0.495 | 2.512 | 0.1911 | 0.577 |
| 001534 | hsa-miR-361-3p | hsa-miR-361-3p, | NA | -0.435 | -1.085 | 0.220 | 0.1923 | 0.577 |
| 001535 | hsa-miR-485-3p | hsa-miR-485-3p, | NA | 1.439 | -0.720 | 3.644 | 0.1927 | 0.577 |
| 001538 | hsa-miR-720 | NA | hsa-miR-720(18), | 0.882 | -0.446 | 2.227 | 0.1938 | 0.5771 |
| 001539 | hsa-miR-891b | hsa-miR-891b, | mml-miR-891(18) | -0.488 | -1.226 | 0.256 | 0.1976 | 0.5825 |
| 001543 | hsa-miR-939 | hsa-miR-939-5p, | hsa-miR-939(18) | 0.540 | -0.281 | 1.368 | 0.1979 | 0.5825 |
| 001545 | hsa-miR-145# | hsa-miR-145-3p, | hsa-miR-145*(17) | -0.711 | -1.794 | 0.383 | 0.2013 | 0.5854 |
| 001546 | hsa-miR-642 | hsa-miR-642a-5p, | hsa-miR-642(15),hsa-miR-642a(17) | 1.239 | -0.657 | 3.172 | 0.2014 | 0.5854 |
| 001551 | hsa-miR-520D-3P | hsa-miR-520d-3p, | NA | -1.496 | -3.772 | 0.835 | 0.2063 | 0.5854 |
| 001553 | hsa-miR-616 | hsa-miR-616-5p | hsa-miR-616(9.2),hsa-miR-616*(17) | -0.520 | -1.331 | 0.298 | 0.2115 | 0.5854 |
| 001555 | hsa-miR-183 | hsa-miR-183-5p, | hsa-miR-183(17), | -0.417 | -1.069 | 0.240 | 0.2126 | 0.5854 |
| 001557 | hsa-miR-10b# | hsa-miR-10b-3p | hsa-miR-10b*(17) | 1.155 | -0.674 | 3.016 | 0.2170 | 0.5854 |
| 001558 | hsa-miR-518b | hsa-miR-518b, | NA | -0.927 | -2.384 | 0.552 | 0.2175 | 0.5854 |
| 001559 | hsa-miR-500 | hsa-miR-500a-3p | hsa-miR-500(9.2),hsa-miR-500*(15),hsa-miR-500a*(17) | -0.615 | -1.585 | 0.365 | 0.2177 | 0.5854 |
| 001560 | hsa-miR-191# | hsa-miR-191-3p, | hsa-miR-191*(17) | -0.577 | -1.489 | 0.343 | 0.2180 | 0.5854 |
| 001562 | hsa-miR-627 | hsa-miR-627-5p, | hsa-miR-627(19) | -0.937 | -2.413 | 0.561 | 0.2186 | 0.5854 |
| 001563 | hsa-miR-1255B | hsa-miR-1255b-5p, | hsa-miR-1255b(17) | -0.681 | -1.756 | 0.407 | 0.2186 | 0.5854 |
| 001566 | hsa-miR-301b | hsa-miR-301b-3p, | hsa-miR-301b(20) | 0.933 | -0.561 | 2.449 | 0.2218 | 0.5854 |
| 001567 | hsa-miR-1180 | hsa-miR-1180-3p, | hsa-miR-1180(19) | -0.576 | -1.497 | 0.352 | 0.2227 | 0.5854 |
| 001568 | hsa-miR-15a | hsa-miR-15a-5p, | hsa-miR-15a(17), | 1.626 | -0.990 | 4.312 | 0.2250 | 0.5854 |
| 001578 | hsa-miR-487b | hsa-miR-487b-3p, | hsa-miR-487b(19), | 1.115 | -0.697 | 2.960 | 0.2289 | 0.5854 |
| 001582 | hsa-miR-140-3p | hsa-miR-140-3p, |  | 1.073 | -0.670 | 2.846 | 0.2289 | 0.5854 |
| 001584 | hsa-miR-30e-3p | hsa-miR-30e-3p, | hsa-miR-30e*(17), | 1.394 | -0.872 | 3.712 | 0.2296 | 0.5854 |
| 001585 | hsa-let-7i# | hsa-let-7i-3p, | hsa-let-7i*(17), | -0.586 | -1.539 | 0.376 | 0.2311 | 0.5854 |
| 001586 | hsa-miR-99a | hsa-miR-99a-5p, | hsa-miR-99a(17), | 1.335 | -0.844 | 3.561 | 0.2314 | 0.5854 |
| 001589 | hsa-miR-192 | hsa-miR-192-5p, | hsa-miR-192(17), | 1.303 | -0.830 | 3.481 | 0.2326 | 0.5854 |
| 001590 | hsa-miR-483-3p | hsa-miR-483-3p | NA | 0.493 | -0.319 | 1.311 | 0.2345 | 0.5854 |
| 001591 | hsa-miR-663B | hsa-miR-663b, | NA | -0.576 | -1.524 | 0.382 | 0.2374 | 0.5854 |
| 001592 | hsa-miR-592 | hsa-miR-592, | mml-miR-592(19) | -0.592 | -1.570 | 0.395 | 0.2383 | 0.5854 |
| 001593 | hsa-let-7e | hsa-let-7e-5p, | hsa-let-7e(17), | 1.627 | -1.099 | 4.428 | 0.2444 | 0.5854 |
| 001597 | hsa-miR-513-5p | hsa-miR-513a-5p, | hsa-miR-513-5p(10) | -0.546 | -1.465 | 0.381 | 0.2471 | 0.5854 |
| 001599 | hsa-miR-220c | NA | hsa-miR-220c(15), | -0.554 | -1.486 | 0.387 | 0.2471 | 0.5854 |
| 001601 | hsa-miR-1184 | hsa-miR-1184, | NA | -0.549 | -1.473 | 0.383 | 0.2471 | 0.5854 |
| 001602 | hsa-miR-384 | hsa-miR-384, | NA | -0.560 | -1.503 | 0.391 | 0.2472 | 0.5854 |
| 001603 | hsa-miR-578 | hsa-miR-578, | NA | -0.561 | -1.504 | 0.392 | 0.2472 | 0.5854 |
| 001604 | hsa-miR-30c-1# | hsa-miR-30c-1-3p, | hsa-miR-30c-1*(17), | -0.563 | -1.510 | 0.393 | 0.2472 | 0.5854 |
| 001605 | hsa-miR-876-5p | hsa-miR-876-5p, | NA | -0.561 | -1.504 | 0.392 | 0.2472 | 0.5854 |
| 001606 | hsa-miR-18b# | hsa-miR-18b-3p | hsa-miR-18b*(17) | -0.559 | -1.499 | 0.390 | 0.2472 | 0.5854 |
| 001607 | hsa-miR-452# | hsa-miR-452-3p | hsa-miR-452*(17) | -0.560 | -1.503 | 0.391 | 0.2472 | 0.5854 |
| 001608 | hsa-let-7c# | NA | hsa-let-7c*(16) | -0.559 | -1.501 | 0.391 | 0.2472 | 0.5854 |
| 001610 | hsa-miR-1248 | hsa-miR-1248, | NA | -0.560 | -1.501 | 0.391 | 0.2472 | 0.5854 |
| 001611 | hsa-miR-488 | hsa-miR-488-3p, | hsa-miR-488(17), | -0.571 | -1.532 | 0.399 | 0.2474 | 0.5854 |
| 001612 | hsa-miR-517# | hsa-miR-517-5p, | hsa-miR-517*(17) | -0.577 | -1.549 | 0.404 | 0.2475 | 0.5854 |
| 001613 | hsa-miR-626 | hsa-miR-626, | NA | -0.577 | -1.549 | 0.404 | 0.2475 | 0.5854 |
| 001614 | hsa-miR-613 | hsa-miR-613, | NA | -0.577 | -1.549 | 0.404 | 0.2475 | 0.5854 |
| 001619 | hsa-miR-524-5p | hsa-miR-524-5p, | hsa-miR-524*(9.2) | -0.577 | -1.549 | 0.404 | 0.2475 | 0.5854 |
| 001621 | hsa-miR-129-3p | hsa-miR-129-2-3p, | hsa-miR-129-3p(17), | -0.579 | -1.552 | 0.405 | 0.2476 | 0.5854 |
| 001624 | hsa-miR-617 | hsa-miR-617, | NA | -0.579 | -1.552 | 0.405 | 0.2476 | 0.5854 |
| 001625 | hsa-miR-941 | hsa-miR-941 | NA | -0.579 | -1.554 | 0.405 | 0.2476 | 0.5854 |
| 001630 | hsa-miR-499-3p | hsa-miR-499a-3p, | hsa-miR-499-3p(17), | -0.583 | -1.564 | 0.408 | 0.2477 | 0.5854 |
| 001663 | hsa-miR-30d# | hsa-miR-30d-3p, | hsa-miR-30d*(17), | 0.723 | -0.512 | 1.974 | 0.2519 | 0.591 |
| 001818 | hsa-miR-889 | hsa-miR-889-3p, | hsa-miR-889(19), | 0.959 | -0.679 | 2.623 | 0.2523 | 0.591 |
| 001821 | hsa-miR-19b-1# | hsa-miR-19b-1-5p, | hsa-miR-19b-1*(17), | 0.436 | -0.319 | 1.197 | 0.2583 | 0.6023 |
| 001823 | hsa-miR-652 | hsa-miR-652-3p, | hsa-miR-652(17), | 1.269 | -0.964 | 3.552 | 0.2671 | 0.6183 |
| 001953 | hsa-miR-331 | hsa-miR-331-3p, | hsa-miR-331(9.2), | 0.731 | -0.559 | 2.038 | 0.2675 | 0.6183 |
| 001979 | hsa-miR-329 | hsa-miR-329-3p, | hsa-miR-329(19), | 0.516 | -0.398 | 1.439 | 0.2689 | 0.6188 |
| 001982 | hsa-miR-376b | hsa-miR-376b-3p, | hsa-miR-376b(18), | 0.905 | -0.713 | 2.550 | 0.2741 | 0.6281 |
| 001984 | hsa-miR-802 | hsa-miR-802, | NA | -0.645 | -1.802 | 0.526 | 0.2788 | 0.6361 |
| 001986 | hsa-miR-18b | hsa-miR-18b-5p, | hsa-miR-18b(17) | -0.718 | -2.026 | 0.608 | 0.2865 | 0.6509 |
| 001988 | hsa-miR-548d-5p | hsa-miR-548d-5p | NA | -0.472 | -1.341 | 0.404 | 0.2894 | 0.6536 |
| 001990 | hsa-miR-886-3p | NA | hsa-miR-886-3p(15), | 0.971 | -0.837 | 2.812 | 0.2943 | 0.6536 |
| 001992 | hsa-miR-744 | hsa-miR-744-5p, | hsa-miR-744(17), | 0.848 | -0.745 | 2.466 | 0.2982 | 0.6536 |
| 001996 | hsa-miR-1249 | hsa-miR-1249-3p, | hsa-miR-1249(20), | -0.322 | -0.931 | 0.290 | 0.3012 | 0.6536 |
| 001998 | hsa-miR-590-5p | hsa-miR-590-5p, | hsa-miR-590(9.2) | 0.792 | -0.715 | 2.321 | 0.3044 | 0.6536 |
| 002002 | hsa-let-7b# | hsa-let-7b-3p, | hsa-let-7b*(17), | -0.491 | -1.423 | 0.451 | 0.3053 | 0.6536 |
| 002004 | hsa-miR-571 | hsa-miR-571, | NA | -0.496 | -1.438 | 0.456 | 0.3055 | 0.6536 |
| 002021 | hsa-miR-567 | hsa-miR-567, | NA | -0.508 | -1.475 | 0.468 | 0.3061 | 0.6536 |
| 002083 | hsa-miR-432# | hsa-miR-432-3p | hsa-miR-432*(17) | -0.509 | -1.476 | 0.469 | 0.3062 | 0.6536 |
| 002084 | hsa-miR-106a# | hsa-miR-106a-3p | hsa-miR-106a*(17) | -0.509 | -1.478 | 0.469 | 0.3062 | 0.6536 |
| 002087 | hsa-miR-92a-1# | hsa-miR-92a-1-5p, | hsa-miR-92a-1*(17) | -0.519 | -1.509 | 0.480 | 0.3068 | 0.6536 |
| 002088 | hsa-miR-450b-3p | hsa-miR-450b-3p, | NA | -0.519 | -1.508 | 0.480 | 0.3068 | 0.6536 |
| 002089 | hsa-miR-132# | hsa-miR-132-5p, | hsa-miR-132*(17), | -0.520 | -1.512 | 0.481 | 0.3069 | 0.6536 |
| 002090 | hsa-miR-1304 | hsa-miR-1304-5p | hsa-miR-1304(17) | -0.521 | -1.513 | 0.481 | 0.3069 | 0.6536 |
| 002092 | hsa-miR-629 | hsa-miR-629-3p | hsa-miR-629(9.2),hsa-miR-629*(17) | 0.792 | -0.725 | 2.332 | 0.3074 | 0.6536 |
| 002093 | hsa-miR-299-5p | hsa-miR-299-5p, | hsa-miR-299(6), | -0.392 | -1.142 | 0.363 | 0.3076 | 0.6536 |
| 002094 | hsa-miR-454# | hsa-miR-454-5p | hsa-miR-454*(17) | -0.567 | -1.652 | 0.530 | 0.3096 | 0.6542 |
| 002096 | hsa-miR-598 | hsa-miR-598-3p, | hsa-miR-598(19), | 0.818 | -0.758 | 2.420 | 0.3103 | 0.6542 |
| 002097 | hsa-miR-520e | hsa-miR-520e, | NA | -2.110 | -6.189 | 2.145 | 0.3255 | 0.6693 |
| 002098 | hsa-miR-452 | hsa-miR-452-5p, | hsa-miR-452(17), | 0.576 | -0.573 | 1.737 | 0.3266 | 0.6693 |
| 002099 | hsa-miR-511 | hsa-miR-511-5p, | hsa-miR-511(19) | 0.616 | -0.614 | 1.861 | 0.3276 | 0.6693 |
| 002100 | hsa-miR-576-5p | hsa-miR-576-5p | NA | -0.271 | -0.811 | 0.273 | 0.3281 | 0.6693 |
| 002102 | hsa-miR-451 | hsa-miR-451a, | hsa-miR-451(17), | -1.568 | -4.703 | 1.671 | 0.3382 | 0.6693 |
| 002105 | hsa-miR-378 | hsa-miR-378a-5p, | hsa-miR-378(9.2),hsa-miR-378*(17), | 1.131 | -1.176 | 3.491 | 0.3390 | 0.6693 |
| 002107 | hsa-miR-383 | hsa-miR-383-5p, | hsa-miR-383(19), | -0.288 | -0.875 | 0.303 | 0.3391 | 0.6693 |
| 002108 | hsa-miR-337-3p | hsa-miR-337-3p, | NA | -0.452 | -1.376 | 0.480 | 0.3401 | 0.6693 |
| 002109 | hsa-miR-656 | hsa-miR-656-3p, | hsa-miR-656(19), | -0.430 | -1.311 | 0.458 | 0.3409 | 0.6693 |
| 002112 | hsa-miR-148a | hsa-miR-148a-3p, | hsa-miR-148(3),hsa-miR-148a(17), | -1.662 | -5.042 | 1.838 | 0.3470 | 0.6693 |
| 002113 | hsa-miR-199a-3p | hsa-miR-199a-3p,hsa-miR-199b-3p, |  | 0.858 | -0.928 | 2.675 | 0.3482 | 0.6693 |
| 002114 | hsa-miR-130b# | hsa-miR-130b-5p | hsa-miR-130b*(17) | -0.535 | -1.647 | 0.589 | 0.3491 | 0.6693 |
| 002115 | hsa-miR-30a-5p | hsa-miR-30a-5p, | hsa-miR-30a(17), | 1.122 | -1.218 | 3.517 | 0.3498 | 0.6693 |
| 002116 | hsa-miR-324-5p | hsa-miR-324-5p, | NA | 0.887 | -0.966 | 2.775 | 0.3500 | 0.6693 |
| 002117 | hsa-miR-422a | hsa-miR-422a, | NA | 0.602 | -0.659 | 1.879 | 0.3502 | 0.6693 |
| 002122 | hsa-miR-301 | hsa-miR-301a-3p, | hsa-miR-301(9.2),hsa-miR-301a(17), | 0.719 | -0.786 | 2.248 | 0.3505 | 0.6693 |
| 002125 | hsa-miR-99b | hsa-miR-99b-5p, | hsa-miR-99b(17), | 0.648 | -0.709 | 2.022 | 0.3506 | 0.6693 |
| 002129 | hsa-miR-144# | hsa-miR-144-5p | hsa-miR-144*(17) | 1.113 | -1.218 | 3.499 | 0.3517 | 0.6693 |
| 002130 | hsa-miR-431 | hsa-miR-431-5p, | hsa-miR-431(17), | 0.524 | -0.587 | 1.647 | 0.3562 | 0.6693 |
| 002132 | hsa-miR-380-5p | hsa-miR-380-5p | hsa-miR-380*(17), | -0.420 | -1.309 | 0.477 | 0.3568 | 0.6693 |
| 002136 | hsa-miR-219-2-3p | hsa-miR-219a-2-3p, | hsa-miR-219-2-3p(19), | -0.420 | -1.307 | 0.476 | 0.3568 | 0.6693 |
| 002137 | hsa-miR-1200 | hsa-miR-1200, | NA | -0.422 | -1.314 | 0.479 | 0.3569 | 0.6693 |
| 002138 | hsa-miR-218-1# | hsa-miR-218-1-3p | hsa-miR-218-1*(17) | -0.424 | -1.320 | 0.481 | 0.3571 | 0.6693 |
| 002139 | hsa-miR-196a# | hsa-miR-196a-3p | hsa-miR-196a*(17) | -0.432 | -1.349 | 0.493 | 0.3579 | 0.6693 |
| 002141 | hsa-miR-744# | hsa-miR-744-3p, | hsa-miR-744*(17), | 0.618 | -0.697 | 1.951 | 0.3581 | 0.6693 |
| 002147 | hsa-miR-92a-2# | hsa-miR-92a-2-5p | hsa-miR-92a-2*(17) | -0.445 | -1.390 | 0.510 | 0.3591 | 0.6693 |
| 002148 | hsa-miR-1275 | hsa-miR-1275, | NA | -0.445 | -1.391 | 0.510 | 0.3592 | 0.6693 |
| 002149 | hsa-miR-29a# | hsa-miR-29a-5p, | hsa-miR-29a*(17), | 0.758 | -0.858 | 2.400 | 0.3593 | 0.6693 |
| 002156 | hsa-miR-871 | mmu-miR-871-5p | hsa-miR-871(10), | -0.448 | -1.399 | 0.513 | 0.3594 | 0.6693 |
| 002157 | hsa-miR-518f# | hsa-miR-518f-5p | hsa-miR-518f*(17) | -0.455 | -1.422 | 0.523 | 0.3602 | 0.6693 |
| 002158 | hsa-miR-524 | hsa-miR-524-3p, | hsa-miR-524(9.2) | -0.455 | -1.424 | 0.524 | 0.3603 | 0.6693 |
| 002159 | hsa-miR-1278 | hsa-miR-1278 | NA | -0.455 | -1.425 | 0.524 | 0.3603 | 0.6693 |
| 002160 | hsa-miR-508-5p | hsa-miR-508-5p | NA | -0.459 | -1.436 | 0.528 | 0.3607 | 0.6693 |
| 002161 | hsa-miR-27a# | hsa-miR-27a-5p, | hsa-miR-27a*(17), | 0.722 | -0.824 | 2.291 | 0.3614 | 0.6693 |
| 002166 | hsa-miR-195# | hsa-miR-195-3p, | hsa-miR-195*(17), | -0.469 | -1.471 | 0.543 | 0.3619 | 0.6693 |
| 002169 | hsa-miR-205 | hsa-miR-205-5p, | hsa-miR-205(17), | -0.607 | -1.917 | 0.721 | 0.3680 | 0.678 |
| 002170 | hsa-miR-331-5p | hsa-miR-331-5p, | NA | 0.481 | -0.575 | 1.548 | 0.3730 | 0.6816 |
| 002172 | hsa-miR-95 | hsa-miR-95-3p, | hsa-miR-95(19), | 0.914 | -1.091 | 2.959 | 0.3738 | 0.6816 |
| 002173 | hsa-miR-34a | hsa-miR-34a-5p, | hsa-miR-34a(17), | 2.087 | -2.469 | 6.856 | 0.3747 | 0.6816 |
| 002174 | hsa-miR-561 | hsa-miR-561-3p | hsa-miR-561(17) | -0.547 | -1.750 | 0.671 | 0.3763 | 0.6816 |
| 002182 | hsa-miR-624 | hsa-miR-624-5p | hsa-miR-624(9.2),hsa-miR-624*(17) | -0.613 | -1.962 | 0.755 | 0.3775 | 0.6816 |
| 002183 | hsa-miR-345 | hsa-miR-345-5p, | hsa-miR-345(17) | 0.703 | -0.856 | 2.286 | 0.3785 | 0.6816 |
| 002184 | hsa-miR-589 | hsa-miR-589-3p | hsa-miR-589(9.2),hsa-miR-589*(17) | -0.359 | -1.156 | 0.444 | 0.3794 | 0.6816 |
| 002185 | hsa-miR-543 | hsa-miR-543, |  | 0.658 | -0.808 | 2.145 | 0.3806 | 0.6816 |
| 002186 | hsa-miR-208 | hsa-miR-208a-3p, | hsa-miR-208(10),hsa-miR-208a(19), | -0.332 | -1.072 | 0.414 | 0.3815 | 0.6816 |
| 002187 | hsa-miR-199a | hsa-miR-199a-5p, | hsa-miR-199a(9.2), | 0.838 | -1.048 | 2.760 | 0.3857 | 0.6867 |
| 002188 | hsa-miR-886-5p | NA | hsa-miR-886-5p(15), | 0.688 | -0.872 | 2.271 | 0.3890 | 0.6902 |
| 002189 | hsa-miR-21 | hsa-miR-21-5p, | hsa-miR-21(17), | -1.132 | -3.670 | 1.473 | 0.3903 | 0.6902 |
| 002193 | hsa-miR-24-2# | hsa-miR-24-2-5p | hsa-miR-24-2*(17) | 0.634 | -0.827 | 2.115 | 0.3965 | 0.6945 |
| 002194 | hsa-miR-215 | hsa-miR-215-5p, | hsa-miR-215(19), | -1.092 | -3.578 | 1.458 | 0.3972 | 0.6945 |
| 002196 | hsa-miR-1296 | hsa-miR-1296-5p, | hsa-miR-1296(19) | -0.235 | -0.776 | 0.310 | 0.3973 | 0.6945 |
| 002198 | hsa-miR-377 | hsa-miR-377-3p, | hsa-miR-377(17), | -0.234 | -0.774 | 0.310 | 0.3980 | 0.6945 |
| 002199 | hsa-miR-19b | hsa-miR-19b-3p, | hsa-miR-19b(17), | 0.488 | -0.662 | 1.651 | 0.4066 | 0.7072 |
| 002201 | hsa-miR-509-5p | hsa-miR-509-5p, | eca-miR-509-5p(20) | -0.423 | -1.424 | 0.588 | 0.4100 | 0.7108 |
| 002202 | hsa-miR-20a | hsa-miR-20a-5p, | hsa-miR-20(6),hsa-miR-20a(17), | -0.554 | -1.872 | 0.783 | 0.4145 | 0.7162 |
| 002203 | hsa-miR-134 | hsa-miR-134-5p, | hsa-miR-134(19), | 0.730 | -1.040 | 2.531 | 0.4207 | 0.7238 |
| 002205 | hsa-miR-23b | hsa-miR-23b-3p, | hsa-miR-23b(17), | 0.564 | -0.807 | 1.953 | 0.4217 | 0.7238 |
| 002208 | hsa-let-7a | hsa-let-7a-5p, | hsa-let-7a(17), | -1.793 | -6.053 | 2.661 | 0.4236 | 0.7247 |
| 002210 | hsa-miR-1285 | hsa-miR-1285-3p, | hsa-miR-1285(17) | -0.218 | -0.753 | 0.320 | 0.4250 | 0.7249 |
| 002211 | hsa-miR-219 | hsa-miR-219a-5p, | hsa-miR-219(9.2),hsa-miR-219-5p(19), | -0.242 | -0.844 | 0.364 | 0.4324 | 0.7351 |
| 002212 | hsa-miR-519a | hsa-miR-519a-3p, | hsa-miR-519a(17) | -0.209 | -0.744 | 0.329 | 0.4455 | 0.7476 |
| 002214 | hsa-miR-411# | hsa-miR-411-3p, | hsa-miR-411*(17), | 0.226 | -0.358 | 0.814 | 0.4481 | 0.7476 |
| 002215 | hsa-miR-335# | hsa-miR-335-3p, | hsa-miR-335*(17) | -0.671 | -2.396 | 1.083 | 0.4504 | 0.7476 |
| 002216 | hsa-miR-641 | hsa-miR-641 | NA | -0.204 | -0.734 | 0.329 | 0.4519 | 0.7476 |
| 002217 | hsa-miR-376a | hsa-miR-376a-3p, | hsa-miR-376a(17), | 1.280 | -2.034 | 4.707 | 0.4531 | 0.7476 |
| 002218 | hsa-let-7d# | hsa-let-7d-3p, | hsa-let-7d*(17), | -0.359 | -1.293 | 0.585 | 0.4547 | 0.7476 |
| 002222 | hsa-miR-1305 | hsa-miR-1305 | NA | -0.206 | -0.743 | 0.335 | 0.4548 | 0.7476 |
| 002227 | hsa-miR-9 | hsa-miR-9-5p, | hsa-miR-9(17), | -0.881 | -3.160 | 1.452 | 0.4556 | 0.7476 |
| 002228 | hsa-miR-544 | hsa-miR-544a, | hsa-miR-544(17) | -0.206 | -0.746 | 0.337 | 0.4565 | 0.7476 |
| 002231 | hsa-miR-361 | hsa-miR-361-5p, | hsa-miR-361(9.2), | 0.719 | -1.173 | 2.648 | 0.4585 | 0.7476 |
| 002233 | hsa-miR-372 | hsa-miR-372-3p, | hsa-miR-372(19), | -0.473 | -1.716 | 0.786 | 0.4592 | 0.7476 |
| 002234 | hsa-miR-141 | hsa-miR-141-3p, | hsa-miR-141(17), | -0.535 | -1.939 | 0.890 | 0.4596 | 0.7476 |
| 002235 | hsa-miR-223 | hsa-miR-223-3p, | hsa-miR-223(17), | 0.373 | -0.621 | 1.377 | 0.4628 | 0.7476 |
| 002237 | hsa-miR-132 | hsa-miR-132-3p, | hsa-miR-132(17), | 1.008 | -1.676 | 3.765 | 0.4651 | 0.7476 |
| 002238 | hsa-miR-517c | hsa-miR-517c-3p, | hsa-miR-517c(17), | -0.453 | -1.671 | 0.781 | 0.4697 | 0.7476 |
| 002239 | hsa-miR-769-5p | hsa-miR-769-5p | NA | 0.551 | -0.937 | 2.060 | 0.4698 | 0.7476 |
| 002240 | hsa-miR-302c | hsa-miR-302c-3p, | hsa-miR-302c(17) | -0.216 | -0.806 | 0.379 | 0.4760 | 0.7476 |
| 002243 | hsa-miR-21# | hsa-miR-21-3p | hsa-miR-21*(17) | -0.209 | -0.783 | 0.368 | 0.4763 | 0.7476 |
| 002244 | hsa-miR-26a-1# | hsa-miR-26a-1-3p, | hsa-miR-26a-1*(17), | -0.438 | -1.638 | 0.776 | 0.4772 | 0.7476 |
| 002245 | hsa-miR-184 | hsa-miR-184, |  | 0.691 | -1.208 | 2.627 | 0.4778 | 0.7476 |
| 002246 | hsa-miR-125b-2# | hsa-miR-125b-2-3p | hsa-miR-125b-2*(17) | -0.206 | -0.772 | 0.364 | 0.4780 | 0.7476 |
| 002247 | hsa-miR-943 | hsa-miR-943, | NA | -0.198 | -0.742 | 0.350 | 0.4780 | 0.7476 |
| 002248 | hsa-miR-17# | hsa-miR-17-3p, | hsa-miR-17*(17), | -0.222 | -0.832 | 0.393 | 0.4783 | 0.7476 |
| 002249 | hsa-miR-9# | hsa-miR-9-3p, | hsa-miR-9*(17), | -0.545 | -2.051 | 0.984 | 0.4819 | 0.7476 |
| 002250 | hsa-miR-629 | hsa-miR-629-5p | hsa-miR-629(17) | -0.403 | -1.521 | 0.727 | 0.4825 | 0.7476 |
| 002251 | hsa-miR-367 | hsa-miR-367-3p, | hsa-miR-367(17), | 1.179 | -2.082 | 4.549 | 0.4827 | 0.7476 |
| 002252 | hsa-miR-137 | hsa-miR-137, |  | -0.192 | -0.728 | 0.347 | 0.4833 | 0.7476 |
| 002253 | hsa-miR-650 | hsa-miR-650, | NA | -0.193 | -0.730 | 0.348 | 0.4835 | 0.7476 |
| 002254 | hsa-miR-423-5p | hsa-miR-423-5p, | NA | -0.531 | -2.008 | 0.969 | 0.4853 | 0.7476 |
| 002255 | hsa-miR-512-3p | hsa-miR-512-3p, | NA | 0.657 | -1.185 | 2.533 | 0.4867 | 0.7476 |
| 002257 | hsa-miR-32 | hsa-miR-32-5p, | hsa-miR-32(17), | 0.572 | -1.043 | 2.213 | 0.4894 | 0.7476 |
| 002258 | hsa-miR-1302 | hsa-miR-1302, | NA | -0.208 | -0.803 | 0.390 | 0.4933 | 0.7476 |
| 002259 | hsa-miR-24 | hsa-miR-24-3p, | hsa-miR-24(17), | 0.395 | -0.733 | 1.537 | 0.4935 | 0.7476 |
| 002260 | hsa-miR-222 | hsa-miR-222-3p, | hsa-miR-222(17), | 0.726 | -1.341 | 2.835 | 0.4938 | 0.7476 |
| 002261 | hsa-miR-128a | hsa-miR-128-3p, | hsa-miR-128(19),hsa-miR-128a(10),hsa-miR-128b(10), | 0.613 | -1.137 | 2.394 | 0.4942 | 0.7476 |
| 002263 | hsa-miR-496 | hsa-miR-496, |  | -0.184 | -0.720 | 0.354 | 0.5003 | 0.7476 |
| 002265 | hsa-miR-17 | hsa-miR-17-5p, | hsa-miR-17(17), | 0.491 | -0.937 | 1.940 | 0.5017 | 0.7476 |
| 002266 | hsa-miR-640 | hsa-miR-640, | NA | -0.185 | -0.725 | 0.357 | 0.5023 | 0.7476 |
| 002267 | hsa-miR-122 | hsa-miR-122-5p, | hsa-miR-122(17), | 0.637 | -1.217 | 2.527 | 0.5027 | 0.7476 |
| 002268 | hsa-miR-337-5p | hsa-miR-337-5p, | NA | 0.480 | -0.920 | 1.899 | 0.5033 | 0.7476 |
| 002269 | hsa-miR-625 | hsa-miR-625-5p | hsa-miR-625(17) | -0.539 | -2.118 | 1.066 | 0.5076 | 0.7476 |
| 002270 | hsa-miR-214 | hsa-miR-214-3p, | hsa-miR-214(17), | 0.853 | -1.655 | 3.424 | 0.5081 | 0.7476 |
| 002271 | hsa-miR-1183 | hsa-miR-1183, | NA | -0.299 | -1.182 | 0.592 | 0.5088 | 0.7476 |
| 002272 | hsa-miR-616 | hsa-miR-616-3p, | hsa-miR-616(17) | -0.200 | -0.792 | 0.396 | 0.5101 | 0.7476 |
| 002275 | hsa-miR-213 | hsa-miR-181a-3p, | hsa-miR-181a*(17),hsa-miR-213(8), | 0.431 | -0.853 | 1.731 | 0.5120 | 0.7476 |
| 002276 | hsa-miR-532-3p | hsa-miR-532-3p, | NA | 0.529 | -1.050 | 2.133 | 0.5129 | 0.7476 |
| 002277 | hsa-miR-449b | hsa-miR-449b-5p, | hsa-miR-449b(17), | -0.202 | -0.804 | 0.405 | 0.5133 | 0.7476 |
| 002278 | hsa-miR-342-5p | hsa-miR-342-5p, | NA | -0.183 | -0.731 | 0.368 | 0.5144 | 0.7476 |
| 002279 | hsa-miR-892b | hsa-miR-892b, | NA | -0.177 | -0.712 | 0.360 | 0.5174 | 0.7476 |
| 002281 | hsa-miR-194 | hsa-miR-194-5p, | hsa-miR-194(17), | 0.758 | -1.540 | 3.109 | 0.5208 | 0.7476 |
| 002282 | hsa-miR-661 | hsa-miR-661 | NA | -0.221 | -0.895 | 0.457 | 0.5214 | 0.7476 |
| 002283 | hsa-miR-589 | hsa-miR-589-5p, | hsa-miR-589(17) | -0.174 | -0.706 | 0.360 | 0.5214 | 0.7476 |
| 002284 | hsa-miR-10b | hsa-miR-10b-5p, | hsa-miR-10b(17), | 0.828 | -1.694 | 3.415 | 0.5229 | 0.7476 |
| 002285 | hsa-miR-504 | hsa-miR-504-5p, | hsa-miR-504(19), | -0.175 | -0.713 | 0.365 | 0.5233 | 0.7476 |
| 002289 | hsa-miR-577 | hsa-miR-577, | mml-miR-577(19) | -0.173 | -0.706 | 0.363 | 0.5250 | 0.7476 |
| 002295 | hsa-miR-221# | hsa-miR-221-5p, | hsa-miR-221*(17) | -0.173 | -0.705 | 0.363 | 0.5258 | 0.7476 |
| 002296 | hsa-let-7f-2# | hsa-let-7f-2-3p, | hsa-let-7f-2*(17) | -0.172 | -0.704 | 0.364 | 0.5284 | 0.7476 |
| 002297 | hsa-miR-541 | hsa-miR-541-3p | hsa-miR-541(17) | -0.171 | -0.704 | 0.364 | 0.5295 | 0.7476 |
| 002299 | hsa-miR-186# | hsa-miR-186-3p | hsa-miR-186*(17) | -0.171 | -0.704 | 0.365 | 0.5304 | 0.7476 |
| 002300 | hsa-miR-25# | hsa-miR-25-5p | hsa-miR-25*(17) | -0.178 | -0.732 | 0.380 | 0.5304 | 0.7476 |
| 002301 | hsa-miR-875-5p | hsa-miR-875-5p, | NA | -0.199 | -0.820 | 0.426 | 0.5316 | 0.7476 |
| 002302 | hsa-miR-218 | hsa-miR-218-5p, | hsa-miR-218(17), | 0.468 | -0.996 | 1.954 | 0.5325 | 0.7476 |
| 002303 | hsa-miR-519d | hsa-miR-519d-3p, | hsa-miR-519d(19) | -0.171 | -0.706 | 0.368 | 0.5333 | 0.7476 |
| 002304 | hsa-miR-605 | hsa-miR-605-5p, | hsa-miR-605(19) | -0.171 | -0.708 | 0.369 | 0.5340 | 0.7476 |
| 002305 | hsa-miR-7-2# | hsa-miR-7-2-3p | hsa-miR-7-2*(17) | -0.170 | -0.704 | 0.368 | 0.5347 | 0.7476 |
| 002306 | hsa-miR-92a | hsa-miR-92a-3p, | hsa-miR-92(5),hsa-miR-92a(17), | -0.411 | -1.702 | 0.897 | 0.5358 | 0.7476 |
| 002308 | hsa-miR-106a | hsa-miR-106a-5p, | hsa-miR-106a(17), | 0.349 | -0.755 | 1.466 | 0.5362 | 0.7476 |
| 002309 | hsa-miR-548b-5p | hsa-miR-548b-5p | NA | -0.532 | -2.205 | 1.171 | 0.5376 | 0.7476 |
| 002310 | hsa-miR-515-3p | hsa-miR-515-3p, | NA | 0.356 | -0.776 | 1.501 | 0.5386 | 0.7476 |
| 002313 | hsa-miR-662 | hsa-miR-662, | NA | -0.175 | -0.732 | 0.385 | 0.5390 | 0.7476 |
| 002314 | hsa-miR-302d | hsa-miR-302d-3p, | hsa-miR-302d(17), | -0.168 | -0.705 | 0.373 | 0.5420 | 0.7496 |
| 002315 | hsa-miR-380-3p | hsa-miR-380-3p, | hsa-miR-380(17) | -0.166 | -0.702 | 0.373 | 0.5458 | 0.7511 |
| 002316 | hsa-miR-1270 | hsa-miR-1270 | NA | -0.166 | -0.703 | 0.375 | 0.5468 | 0.7511 |
| 002317 | hsa-miR-525 | hsa-miR-525-5p, | hsa-miR-525(9.2) | -0.235 | -0.998 | 0.534 | 0.5473 | 0.7511 |
| 002322 | hsa-miR-122# | hsa-miR-122-3p, | hsa-miR-122*(17) | -0.168 | -0.719 | 0.387 | 0.5523 | 0.756 |
| 002323 | hsa-miR-654 | hsa-miR-654-5p, | hsa-miR-654(9.2) | -0.339 | -1.468 | 0.802 | 0.5584 | 0.7607 |
| 002324 | hsa-miR-432 | hsa-miR-432-5p, | hsa-miR-432(17), | 0.470 | -1.105 | 2.070 | 0.5603 | 0.7607 |
| 002325 | hsa-miR-193b# | hsa-miR-193b-5p, | hsa-miR-193b*(17), | -0.160 | -0.697 | 0.381 | 0.5614 | 0.7607 |
| 002326 | hsa-miR-888 | hsa-miR-888-5p, | hsa-miR-888(17) | 0.809 | -1.900 | 3.593 | 0.5615 | 0.7607 |
| 002329 | hsa-miR-195 | hsa-miR-195-5p, | hsa-miR-195(17), | 0.489 | -1.180 | 2.185 | 0.5676 | 0.7637 |
| 002330 | hsa-miR-542-3p | hsa-miR-542-3p, | NA | 0.362 | -0.876 | 1.616 | 0.5676 | 0.7637 |
| 002331 | hsa-miR-665 | hsa-miR-665, | NA | -0.158 | -0.701 | 0.388 | 0.5687 | 0.7637 |
| 002332 | hsa-miR-139-3p |  | hsa-miR-139-3p(18) | 0.976 | -2.366 | 4.432 | 0.5712 | 0.7637 |
| 002333 | hsa-miR-147 | hsa-miR-147a, | hsa-miR-147(17), | -0.154 | -0.691 | 0.385 | 0.5741 | 0.7637 |
| 002334 | hsa-miR-1254 | hsa-miR-1254, | NA | -0.152 | -0.684 | 0.382 | 0.5758 | 0.7637 |
| 002336 | hsa-miR-429 | hsa-miR-429, | mml-miR-429(19) | -0.160 | -0.722 | 0.405 | 0.5770 | 0.7637 |
| 002338 | hsa-miR-455-3p | hsa-miR-455-3p, | ssc-miR-455(18) | -0.183 | -0.824 | 0.463 | 0.5778 | 0.7637 |
| 002339 | hsa-miR-210 | hsa-miR-210-3p, | hsa-miR-210(19), | 1.126 | -2.800 | 5.210 | 0.5789 | 0.7637 |
| 002340 | hsa-miR-1179 | hsa-miR-1179, | NA | -0.177 | -0.802 | 0.453 | 0.5808 | 0.7637 |
| 002341 | hsa-miR-564 | hsa-miR-564, | NA | -0.152 | -0.691 | 0.390 | 0.5810 | 0.7637 |
| 002346 | hsa-miR-636 | hsa-miR-636 | NA | -1.288 | -5.744 | 3.379 | 0.5819 | 0.7637 |
| 002347 | hsa-miR-455 | hsa-miR-455-5p, | hsa-miR-455(9.2), | 0.225 | -0.576 | 1.033 | 0.5827 | 0.7637 |
| 002349 | hsa-miR-491-3p | hsa-miR-491-3p, | mmu-miR-491*(17) | -0.150 | -0.688 | 0.391 | 0.5857 | 0.7637 |
| 002350 | hsa-miR-1247 | hsa-miR-1247-5p, | hsa-miR-1247(17), | -0.150 | -0.688 | 0.391 | 0.5862 | 0.7637 |
| 002351 | hsa-miR-885-5p | hsa-miR-885-5p, | NA | 0.664 | -1.732 | 3.119 | 0.5896 | 0.7637 |
| 002352 | hsa-miR-1290 | hsa-miR-1290, | NA | 0.219 | -0.575 | 1.020 | 0.5897 | 0.7637 |
| 002354 | hsa-miR-648 | hsa-miR-648, | NA | -0.184 | -0.853 | 0.488 | 0.5898 | 0.7637 |
| 002355 | hsa-miR-16-1# | hsa-miR-16-1-3p, | hsa-miR-16-1*(17), | 0.339 | -0.899 | 1.593 | 0.5924 | 0.7652 |
| 002357 | hsa-miR-325 | hsa-miR-325, | NA | -0.155 | -0.723 | 0.417 | 0.5949 | 0.7664 |
| 002358 | hsa-miR-34a# | hsa-miR-34a-3p | hsa-miR-34a*(17) | 0.489 | -1.310 | 2.321 | 0.5962 | 0.7664 |
| 002360 | hsa-miR-1301 | hsa-miR-1301-3p, | hsa-miR-1301(19) | -0.146 | -0.693 | 0.404 | 0.6012 | 0.7684 |
| 002361 | hsa-miR-328 | hsa-miR-328-3p, | hsa-miR-328(19), | -0.402 | -1.898 | 1.117 | 0.6016 | 0.7684 |
| 002363 | hsa-miR-520d-5p | hsa-miR-520d-5p, |  | -0.144 | -0.685 | 0.400 | 0.6022 | 0.7684 |
| 002364 | hsa-miR-520f | hsa-miR-520f-3p, | hsa-miR-520f(19), | -0.324 | -1.541 | 0.909 | 0.6047 | 0.7697 |
| 002365 | hsa-miR-26a | hsa-miR-26a-5p, | hsa-miR-26a(17), | 0.375 | -1.072 | 1.844 | 0.6126 | 0.7779 |
| 002366 | hsa-miR-548d | hsa-miR-548d-3p | hsa-miR-548d(9.2) | -0.308 | -1.524 | 0.923 | 0.6217 | 0.7868 |
| 002367 | hsa-miR-148b | hsa-miR-148b-3p, | hsa-miR-148b(17), | 0.483 | -1.428 | 2.430 | 0.6226 | 0.7868 |
| 002369 | hsa-miR-494 | hsa-miR-494-3p, | hsa-miR-494(19), | -0.435 | -2.186 | 1.347 | 0.6294 | 0.7908 |
| 002370 | hsa-miR-625# | hsa-miR-625-3p | hsa-miR-625*(17) | -0.570 | -2.861 | 1.776 | 0.6308 | 0.7908 |
| 002376 | hsa-miR-450a | hsa-miR-450a-5p, | hsa-miR-450a(17), | -0.142 | -0.720 | 0.440 | 0.6320 | 0.7908 |
| 002378 | hsa-miR-668 | hsa-miR-668-3p, | hsa-miR-668(19) | -0.223 | -1.133 | 0.696 | 0.6327 | 0.7908 |
| 002380 | hsa-miR-339-3p | hsa-miR-339-3p, | NA | 0.402 | -1.241 | 2.073 | 0.6332 | 0.7908 |
| 002384 | hsa-miR-342-3p | hsa-miR-342-3p, | NA | 0.962 | -2.980 | 5.064 | 0.6370 | 0.7937 |
| 002386 | hsa-miR-26b# | hsa-miR-26b-3p, | hsa-miR-26b*(17), | 0.271 | -0.924 | 1.481 | 0.6579 | 0.8162 |
| 002387 | hsa-miR-522 | hsa-miR-522-3p, | hsa-miR-522(17), | -0.134 | -0.724 | 0.461 | 0.6585 | 0.8162 |
| 002388 | hsa-miR-145 | hsa-miR-145-5p, | hsa-miR-145(17), | -0.582 | -3.136 | 2.040 | 0.6601 | 0.8162 |
| 002390 | hsa-miR-486 | hsa-miR-486-5p, | hsa-miR-486(9.2), | -0.406 | -2.206 | 1.427 | 0.6614 | 0.8162 |
| 002392 | hsa-miR-330 | hsa-miR-330-3p, | hsa-miR-330(9.2) | -0.547 | -3.023 | 1.991 | 0.6691 | 0.8221 |
| 002393 | hsa-miR-518d | hsa-miR-518d-3p, | hsa-miR-518d(9.2) | -0.232 | -1.292 | 0.839 | 0.6692 | 0.8221 |
| 002397 | hsa-miR-199b | hsa-miR-199b-5p, | hsa-miR-199b(9.2), | -0.385 | -2.147 | 1.408 | 0.6711 | 0.8222 |
| 002398 | hsa-miR-576-3p | hsa-miR-576-3p, | NA | -0.308 | -1.723 | 1.128 | 0.6725 | 0.8222 |
| 002400 | hsa-miR-874 | hsa-miR-874-3p, | hsa-miR-874(19), | -0.136 | -0.780 | 0.511 | 0.6785 | 0.8255 |
| 002402 | hsa-miR-96 | hsa-miR-96-5p, | hsa-miR-96(17), | 0.350 | -1.298 | 2.025 | 0.6787 | 0.8255 |
| 002403 | hsa-miR-1262 | hsa-miR-1262, | NA | -0.170 | -0.975 | 0.641 | 0.6798 | 0.8255 |
| 002404 | hsa-miR-100 | hsa-miR-100-5p, | hsa-miR-100(17), | -0.557 | -3.207 | 2.165 | 0.6848 | 0.8286 |
| 002405 | rno-miR-29c# | hsa-miR-29c-5p, | hsa-miR-29c*(17), | -0.227 | -1.320 | 0.878 | 0.6855 | 0.8286 |
| 002406 | hsa-miR-409-5p | hsa-miR-409-5p, |  | -0.148 | -0.878 | 0.588 | 0.6929 | 0.8356 |
| 002408 | hsa-miR-1265 | hsa-miR-1265, | NA | -0.107 | -0.645 | 0.434 | 0.6972 | 0.8387 |
| 002409 | hsa-miR-483-5p | hsa-miR-483-5p | NA | 0.619 | -2.487 | 3.823 | 0.6995 | 0.8387 |
| 002410 | hsa-miR-375 | hsa-miR-375, |  | 0.356 | -1.444 | 2.188 | 0.7003 | 0.8387 |
| 002413 | hsa-let-7b | hsa-let-7b-5p, | hsa-let-7b(17), | -0.302 | -1.837 | 1.258 | 0.7024 | 0.8393 |
| 002414 | hsa-miR-101 | hsa-miR-101-3p, | hsa-miR-101(17), | 0.308 | -1.290 | 1.932 | 0.7073 | 0.8433 |
| 002415 | hsa-miR-106b# | hsa-miR-106b-3p, | hsa-miR-106b*(17), | -0.352 | -2.191 | 1.521 | 0.7103 | 0.8442 |
| 002417 | hsa-miR-601 | hsa-miR-601, | NA | -0.550 | -3.415 | 2.400 | 0.7112 | 0.8442 |
| 002418 | hsa-miR-584 | hsa-miR-584-5p, | hsa-miR-584(17), | -0.114 | -0.722 | 0.498 | 0.7141 | 0.8456 |
| 002419 | hsa-miR-410 | hsa-miR-410-3p, | hsa-miR-410(19), | -0.336 | -2.147 | 1.507 | 0.7183 | 0.847 |
| 002420 | hsa-miR-27b | hsa-miR-27b-3p, | hsa-miR-27b(17), | -0.312 | -1.998 | 1.403 | 0.7194 | 0.847 |
| 002421 | hsa-miR-942 | hsa-miR-942-5p, | hsa-miR-942(19) | 0.474 | -2.094 | 3.109 | 0.7201 | 0.847 |
| 002422 | hsa-miR-130a | hsa-miR-130a-3p, | hsa-miR-130a(17), | -0.273 | -1.796 | 1.273 | 0.7275 | 0.8538 |
| 002423 | hsa-miR-491 | hsa-miR-491-5p, | mmu-miR-491(17) | -0.380 | -2.513 | 1.799 | 0.7295 | 0.8544 |
| 002425 | hsa-miR-25 | hsa-miR-25-3p, | hsa-miR-25(17), | -0.224 | -1.514 | 1.082 | 0.7347 | 0.8557 |
| 002427 | hsa-miR-374-5p | hsa-miR-374b-5p, | hsa-miR-374b(17), | 0.506 | -2.405 | 3.505 | 0.7359 | 0.8557 |
| 002428 | hsa-miR-136# | hsa-miR-136-3p, | hsa-miR-136*(17), | -0.295 | -1.996 | 1.435 | 0.7361 | 0.8557 |
| 002429 | hsa-miR-548J | hsa-miR-548j-5p | hsa-miR-548j(19) | -0.095 | -0.653 | 0.466 | 0.7386 | 0.8557 |
| 002431 | hsa-miR-133a | hsa-miR-133a-3p, | hsa-miR-133a(19), | -0.380 | -2.591 | 1.881 | 0.7390 | 0.8557 |
| 002432 | hsa-miR-154 | hsa-miR-154-5p, | hsa-miR-154(17), | 0.121 | -0.592 | 0.839 | 0.7404 | 0.8557 |
| 002433 | hsa-miR-152 | hsa-miR-152-3p, | hsa-miR-152(19), | -0.225 | -1.573 | 1.141 | 0.7448 | 0.8586 |
| 002434 | hsa-miR-362-3p | hsa-miR-362-3p, | NA | 0.211 | -1.062 | 1.500 | 0.7462 | 0.8586 |
| 002435 | hsa-miR-154# | hsa-miR-154-3p, | hsa-miR-154*(17), | -0.176 | -1.247 | 0.907 | 0.7490 | 0.8598 |
| 002436 | hsa-miR-206 | hsa-miR-206, |  | -0.254 | -1.815 | 1.331 | 0.7513 | 0.8598 |
| 002437 | hsa-miR-125b | hsa-miR-125b-5p, | hsa-miR-125b(17), | -0.224 | -1.602 | 1.174 | 0.7521 | 0.8598 |
| 002438 | hsa-miR-320B | hsa-miR-320b, | NA | 0.234 | -1.225 | 1.715 | 0.7541 | 0.8602 |
| 002439 | hsa-miR-579 | hsa-miR-579-3p, | hsa-miR-579(19) | -0.206 | -1.522 | 1.127 | 0.7601 | 0.8639 |
| 002441 | hsa-miR-1269 | hsa-miR-1269a | hsa-miR-1269(17) | -0.087 | -0.647 | 0.476 | 0.7606 | 0.8639 |
| 002442 | hsa-miR-193a-3p | hsa-miR-193a-3p, |  | -0.136 | -1.033 | 0.769 | 0.7669 | 0.8688 |
| 002443 | hsa-miR-324-3p | hsa-miR-324-3p, | NA | 0.273 | -1.540 | 2.119 | 0.7692 | 0.8688 |
| 002444 | hsa-miR-7 | hsa-miR-7-5p, | hsa-miR-7(17), | -0.185 | -1.419 | 1.065 | 0.7705 | 0.8688 |
| 002445 | hsa-miR-135b# | hsa-miR-135b-3p, | hsa-miR-135b*(17), | -0.103 | -0.802 | 0.600 | 0.7728 | 0.8688 |
| 002446 | hsa-miR-516b | hsa-miR-516b-5p, | hsa-miR-516-5p(7),hsa-miR-516b(17), | -0.087 | -0.677 | 0.506 | 0.7731 | 0.8688 |
| 002447 | hsa-miR-425-5p | hsa-miR-425-5p, | hsa-miR-425(17), | -0.497 | -3.865 | 2.989 | 0.7763 | 0.8693 |
| 002619 | hsa-miR-505# | hsa-miR-505-5p | hsa-miR-505*(17) | 0.222 | -1.311 | 1.780 | 0.7775 | 0.8693 |
| 002623 | hsa-miR-487a | hsa-miR-487a-3p, | hsa-miR-487(7.1),hsa-miR-487a(19) | -0.122 | -0.980 | 0.744 | 0.7812 | 0.8693 |
| 002642 | hsa-miR-519e | hsa-miR-519e-3p, | hsa-miR-519e(17) | -0.110 | -0.883 | 0.669 | 0.7818 | 0.8693 |
| 002658 | hsa-miR-196b | hsa-miR-196b-5p, | hsa-miR-196b(17), | -0.204 | -1.648 | 1.261 | 0.7834 | 0.8693 |
| 002675 | hsa-miR-99b# | hsa-miR-99b-3p, | hsa-miR-99b*(17), | 0.284 | -1.755 | 2.365 | 0.7866 | 0.8693 |
| 002676 | hsa-miR-212 | hsa-miR-212-3p, | hsa-miR-212(17), | 0.297 | -1.841 | 2.482 | 0.7871 | 0.8693 |
| 002677 | hsa-miR-200a | hsa-miR-200a-3p, | hsa-miR-200a(17), | 0.091 | -0.568 | 0.754 | 0.7880 | 0.8693 |
| 002678 | hsa-miR-502-3p | hsa-miR-502-3p, | NA | -0.170 | -1.406 | 1.081 | 0.7885 | 0.8693 |
| 002681 | hsa-miR-149 | hsa-miR-149-5p, | hsa-miR-149(17), | -0.076 | -0.647 | 0.498 | 0.7940 | 0.8736 |
| 002743 | hsa-miR-501-3p | hsa-miR-501-3p, | NA | -0.117 | -1.013 | 0.787 | 0.7982 | 0.8762 |
| 002758 | hsa-miR-202 | hsa-miR-202-3p, | hsa-miR-202(17) | -0.165 | -1.435 | 1.121 | 0.7997 | 0.8762 |
| 002763 | hsa-miR-148b# | hsa-miR-148b-5p | hsa-miR-148b*(17) | -0.164 | -1.431 | 1.120 | 0.8014 | 0.8762 |
| 002769 | hsa-miR-944 | hsa-miR-944, | NA | -0.072 | -0.651 | 0.510 | 0.8074 | 0.8809 |
| 002776 | hsa-miR-130b | hsa-miR-130b-3p, | hsa-miR-130b(17), | 0.221 | -1.573 | 2.048 | 0.8105 | 0.8826 |
| 002777 | hsa-miR-545 | hsa-miR-545-3p, | hsa-miR-545(17) | 0.130 | -1.000 | 1.273 | 0.8223 | 0.8922 |
| 002779 | hsa-miR-106b | hsa-miR-106b-5p, | hsa-miR-106b(17), | 0.143 | -1.108 | 1.410 | 0.8239 | 0.8922 |
| 002783 | hsa-miR-302b | hsa-miR-302b-3p, | hsa-miR-302b(17), | -0.069 | -0.684 | 0.549 | 0.8257 | 0.8922 |
| 002789 | hsa-miR-660 | hsa-miR-660-5p, | hsa-miR-660(17), | -0.141 | -1.393 | 1.127 | 0.8261 | 0.8922 |
| 002790 | hsa-miR-548c-5p | hsa-miR-548am-5p,hsa-miR-548c-5p,hsa-miR-548o-5p, | NA | -0.102 | -1.041 | 0.846 | 0.8327 | 0.8961 |
| 002791 | hsa-miR-424 | hsa-miR-424-5p, | hsa-miR-424(17), | -0.143 | -1.462 | 1.195 | 0.8332 | 0.8961 |
| 002792 | hsa-miR-591 | hsa-miR-591, | NA | 0.060 | -0.545 | 0.669 | 0.8462 | 0.9082 |
| 002798 | hsa-let-7f | hsa-let-7f-5p, | hsa-let-7f(17), | -0.363 | -4.003 | 3.415 | 0.8481 | 0.9084 |
| 002801 | hsa-miR-365 | hsa-miR-365a-3p,hsa-miR-365b-3p, | hsa-miR-365(17), | 0.169 | -1.603 | 1.973 | 0.8530 | 0.9111 |
| 002807 | hsa-miR-15b# | hsa-miR-15b-3p, | hsa-miR-15b*(17), | 0.167 | -1.598 | 1.963 | 0.8541 | 0.9111 |
| 002818 | hsa-miR-124a | hsa-miR-124-3p, | hsa-miR-124(17), | 0.058 | -0.603 | 0.724 | 0.8637 | 0.9196 |
| 002822 | hsa-miR-30a-3p | hsa-miR-30a-3p, | hsa-miR-30a(3.1),hsa-miR-30a*(17), | 0.193 | -2.075 | 2.513 | 0.8688 | 0.9231 |
| 002824 | hsa-miR-200b | hsa-miR-200b-3p, | hsa-miR-200b(17), | 0.106 | -1.183 | 1.412 | 0.8725 | 0.9252 |
| 002827 | hsa-miR-1226# | hsa-miR-1226-5p | hsa-miR-1226*(17) | 0.046 | -0.544 | 0.640 | 0.8782 | 0.9294 |
| 002829 | hsa-miR-93 | hsa-miR-93-5p, | hsa-miR-93(17), | 0.106 | -1.299 | 1.531 | 0.8829 | 0.9325 |
| 002838 | hsa-miR-500 | hsa-miR-500a-5p, | hsa-miR-500(15),hsa-miR-500a(17), | -0.093 | -1.384 | 1.215 | 0.8883 | 0.9362 |
| 002840 | hsa-miR-646 | hsa-miR-646, | NA | 0.123 | -1.647 | 1.924 | 0.8928 | 0.9391 |
| 002841 | hsa-miR-424# | hsa-miR-424-3p, | hsa-miR-424*(17), | 0.068 | -0.968 | 1.114 | 0.8985 | 0.9424 |
| 002842 | hsa-miR-551b | hsa-miR-551b-3p, | hsa-miR-551b(17), | -0.148 | -2.414 | 2.171 | 0.8995 | 0.9424 |
| 002843 | rno-miR-7# | hsa-miR-7-1-3p, | hsa-miR-7-1*(17), | 0.157 | -2.322 | 2.698 | 0.9025 | 0.9437 |
| 002844 | hsa-miR-18a | hsa-miR-18a-5p, | hsa-miR-18a(17), | -0.083 | -1.504 | 1.359 | 0.9097 | 0.9493 |
| 002847 | hsa-miR-216b | hsa-miR-216b-5p, | hsa-miR-216b(19), | 0.036 | -0.679 | 0.756 | 0.9221 | 0.9604 |
| 002851 | hsa-miR-580 | hsa-miR-580-3p, | hsa-miR-580(19) | -0.027 | -0.598 | 0.547 | 0.9263 | 0.9628 |
| 002852 | hsa-miR-1276 | hsa-miR-1276, | NA | 0.048 | -1.262 | 1.376 | 0.9429 | 0.9782 |
| 002854 | hsa-miR-574-3p | hsa-miR-574-3p, | NA | 0.054 | -1.657 | 1.795 | 0.9512 | 0.9826 |
| 002857 | hsa-miR-382 | hsa-miR-382-5p, | hsa-miR-382(17), | 0.111 | -3.706 | 4.079 | 0.9554 | 0.9826 |
| 002860 | hsa-miR-185 | hsa-miR-185-5p, | hsa-miR-185(17), | -0.037 | -1.344 | 1.286 | 0.9555 | 0.9826 |
| 002863 | hsa-miR-221 | hsa-miR-221-3p, | hsa-miR-221(17), | 0.051 | -1.739 | 1.874 | 0.9556 | 0.9826 |
| 002867 | hsa-miR-489 | hsa-miR-489-3p, | hsa-miR-489(19), | -0.023 | -0.858 | 0.818 | 0.9566 | 0.9826 |
| 002868 | hsa-miR-151-3p | hsa-miR-151a-3p, | hsa-miR-151-3p(17), | 0.087 | -3.195 | 3.481 | 0.9591 | 0.9826 |
| 002870 | hsa-miR-363 | hsa-miR-363-3p, | hsa-miR-363(17), | 0.070 | -2.661 | 2.879 | 0.9602 | 0.9826 |
| 002874 | hsa-miR-654-3p | hsa-miR-654-3p, | NA | 0.014 | -0.563 | 0.594 | 0.9631 | 0.9836 |
| 002880 | hsa-miR-532 | hsa-miR-532-5p, | hsa-miR-532(9.2), | -0.044 | -2.033 | 1.984 | 0.9655 | 0.9841 |
| 002883 | hsa-miR-143 | hsa-miR-143-3p, | hsa-miR-143(17), | -0.030 | -1.605 | 1.569 | 0.9701 | 0.987 |
| 002884 | hsa-miR-674 |  | hsa-miR-674(10), | -0.016 | -1.034 | 1.012 | 0.9756 | 0.9907 |
| 002893 | hsa-miR-144 | hsa-miR-144-3p, | hsa-miR-144(17), | -0.009 | -0.681 | 0.667 | 0.9783 | 0.9914 |
| 002895 | hsa-miR-618 | hsa-miR-618, | NA | -0.040 | -3.347 | 3.381 | 0.9814 | 0.9927 |
| 002896 | hsa-miR-497 | hsa-miR-497-5p, | hsa-miR-497(17), | -0.009 | -0.915 | 0.906 | 0.9848 | 0.9941 |
| 002897 | hsa-miR-374a# | hsa-miR-374a-3p, | hsa-miR-374a*(17) | -0.005 | -0.576 | 0.570 | 0.9875 | 0.9941 |
| 002901 | hsa-miR-671-3p | hsa-miR-671-3p, | NA | 0.010 | -1.339 | 1.378 | 0.9884 | 0.9941 |
| 002902 | hsa-miR-27a | hsa-miR-27a-3p, | hsa-miR-27a(17), | 0.005 | -1.487 | 1.519 | 0.9951 | 0.9977 |
| 002904 | hsa-miR-19a | hsa-miR-19a-3p, | hsa-miR-19a(17), | -0.004 | -1.467 | 1.481 | 0.9958 | 0.9977 |
| 002908 | hsa-miR-193a-5p | hsa-miR-193a-5p, |  | 0.000 | -1.603 | 1.629 | 0.9996 | 0.9996 |

**Supplemental Table 3.** PCSK9 mean concentration levels grouped by LDL cholesterol (≤ 116 mg/dL or >116 mg/dL).

| **N** | **LDL cholesterol** | **PCSK9 (ng/dL)** | **CI 95%** | | **P-value** |
| --- | --- | --- | --- | --- | --- |
| **258 (35.4%)** | **≤ 116 mg/dL** | 269.9 | 258.8 | 281 | 0.0067* |
| **471 (64.6%)** | **> 116 mg/dl** | 290.1 | 280.7 | 299.5 |  |

*p-value from t-test; LDL, low-density lipoprotein; PCSK9, proprotein convertase subtilisin/kexin type 9. PCSK9 values are expressed as mean and 95% CI; n= numerosity.

**Supplemental Table 4.** Association between miRNA levels (RQ) and LDL receptor (RQ) with multivariable linear regression models, grouped by LDL cholesterol (≤ 116, >116 mg/dL)

|  | **LDL cholesterol ≤ 116 mg/dL** | | | | **LDL cholesterol > 116 mg/dL** | | | |
| --- | --- | --- | --- | --- | --- | --- | --- | --- |
|  |  |  |  |  |  |  |  |  |
|  | **β** | **95% CI** | **P-value** | **β** | | **95% CI** | **P-value** |  |
| **hsa-miR-362-5p + has-miR-150 + has-miR-520b-3p + hsa-miR-638** | 0.001 | -0.06 ; 0.06 | 0.9631 | **-0.053** | | **-0.099;-0.008** | **0.0296** |  |
| **Multivariable model:** |  |  |  |  | |  |  |  |
| **hsa-miR-362-5p** | 0.016 | -0.054; 0.086 | 0.6421 | 0.023 | | -0.032; 0.079 | 0.4032 |  |
| **has-miR-150** | -0.004 | -0.075; 0.068 | 0.9159 | **-0.065** | | **-0.118; -0.011** | **0.0182** |  |
| **has-miR-520b-3p** | -0.003 | -0.068; 0.062 | 0.9306 | -0.02 | | -0.067; 0.026 | 0.8502 |  |
| **hsa-miR-638** | -0.008 | -0.006; 0.045 | 0.767 | -0.005 | | -0.057; 0.047 | 0.3925 |  |

All linear regression models were adjusted for age, gender, BMI. Regression coefficients were calculated for 1 standard deviation increase in each predictor variable. β regression coefficients were reported for one SD increment (SD_sum_of_four=2425.44; SD_ hsa-miR-362-5p=1.96; SD_ hsa-miR-150=2424.43; SD_ hsa-miR-638=6.93; SD_ hsa-miR-520b-3p=10.55).

**Supplemental Figure 1**

**
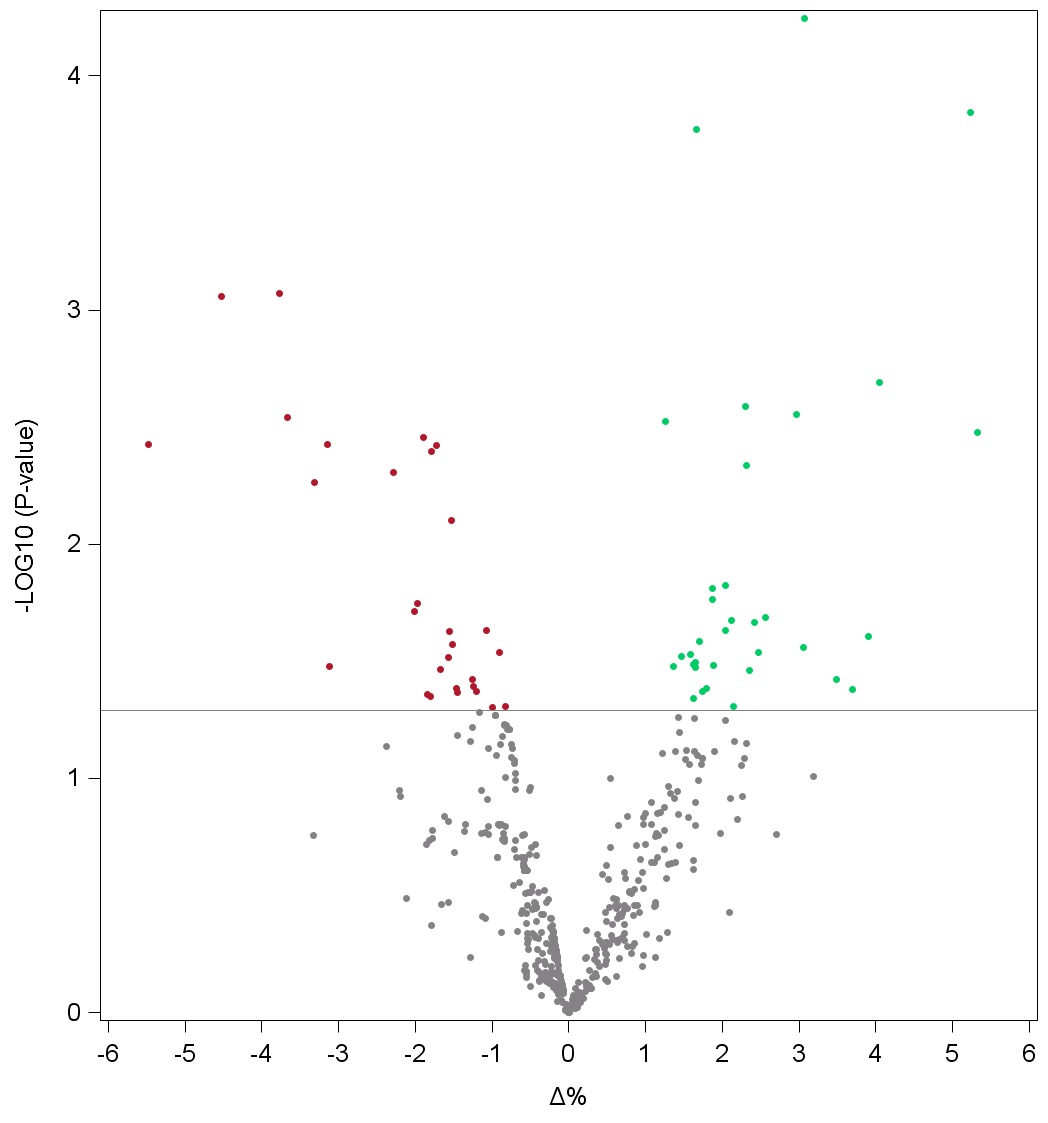
**

**Supplementary legend Figure 1.** *Volcano plot* representing the results of the linear regression models showing the association between EV-miRNAs and PCSK9, adjusted for age, gender, BMI, smoking habit, statin use, PM10, and apparent temperature measured at the day before the blood draw. Each dot represents a miRNA and they are displayed based on the % variation (∆% = (exp(β) − 1) * 100) (x-axis) and the negative logarithm (base 10) of the P-value (y-axis). The grey line represents P-value equal to 0.05. BMI, body mass index; EV, extracellular vesicles; PCSK9), proprotein convertase subtilisin/kexin type 9; PM, particulate matter.


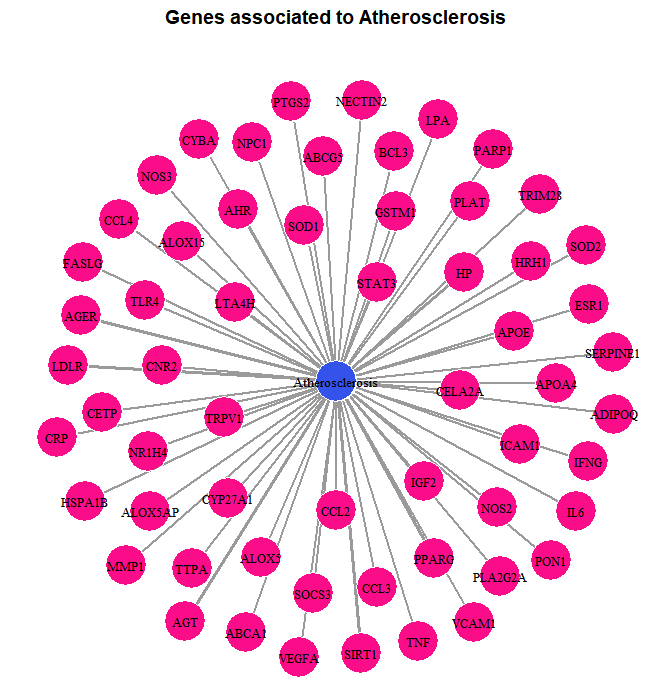
**Supplemental Figure 2**

**Supplementary legend Figure legend 2.** *Maps of genes associated with atherosclerosis*. Network of genes related to atherosclerosis

STROBE Statement—Checklist of items that should be included in reports of ***cohort studies***

|  | Item No | Recommendation | Page No |
| --- | --- | --- | --- |
| **Title and abstract** | 1 | (*a*) Indicate the study’s design with a commonly used term in the title or the abstract | 2 |
|  |  | (*b*) Provide in the abstract an informative and balanced summary of what was done and what was found | 2 |
| Introduction | | | |
| Background/rationale | 2 | Explain the scientific background and rationale for the investigation being reported | 3-4 |
| Objectives | 3 | State specific objectives, including any prespecified hypotheses | 4 |
| Methods | | | |
| Study design | 4 | Present key elements of study design early in the paper | 4 |
| Setting | 5 | Describe the setting, locations, and relevant dates, including periods of recruitment, exposure, follow-up, and data collection | 4 |
| Participants | 6 | (*a*) Give the eligibility criteria, and the sources and methods of selection of participants. Describe methods of follow-up | 4 |
|  |  | (*b*) For matched studies, give matching criteria and number of exposed and unexposed | 4 |
| Variables | 7 | Clearly define all outcomes, exposures, predictors, potential confounders, and effect modifiers. Give diagnostic criteria, if applicable | 7 |
| Data sources/ measurement | 8* | For each variable of interest, give sources of data and details of methods of assessment (measurement). Describe comparability of assessment methods if there is more than one group |  |
| Bias | 9 | Describe any efforts to address potential sources of bias | 7 |
| Study size | 10 | Explain how the study size was arrived at | 7 |
| Quantitative variables | 11 | Explain how quantitative variables were handled in the analyses. If applicable, describe which groupings were chosen and why | 7 |
| Statistical methods | 12 | (*a*) Describe all statistical methods, including those used to control for confounding | 7 |
|  |  | (*b*) Describe any methods used to examine subgroups and interactions | 7 |
|  |  | (*c*) Explain how missing data were addressed | 7 |
|  |  | (*d*) If applicable, explain how loss to follow-up was addressed | 7 |
|  |  | (*e*) Describe any sensitivity analyses | 7 |
| Results | | |  |
| Participants | 13* | (a) Report numbers of individuals at each stage of study—eg numbers potentially eligible, examined for eligibility, confirmed eligible, included in the study, completing follow-up, and analysed | 9 and 21 |
|  |  | (b) Give reasons for non-participation at each stage | 9 |
|  |  | (c) Consider use of a flow diagram | - |
| Descriptive data | 14* | (a) Give characteristics of study participants (eg demographic, clinical, social) and information on exposures and potential confounders | 9 |
|  |  | (b) Indicate number of participants with missing data for each variable of interest | 9 |
|  |  | (c) Summarise follow-up time (eg, average and total amount) | 9 |
| Outcome data | 15* | Report numbers of outcome events or summary measures over time | - |

| Main results | 16 | (*a*) Give unadjusted estimates and, if applicable, confounder-adjusted estimates and their precision (eg, 95% confidence interval). Make clear which confounders were adjusted for and why they were included | 10-11 and 22 |
| --- | --- | --- | --- |
|  |  | (*b*) Report category boundaries when continuous variables were categorized | 22-23 |
|  |  | (*c*) If relevant, consider translating estimates of relative risk into absolute risk for a meaningful time period | - |
| Other analyses | 17 | Report other analyses done—eg analyses of subgroups and interactions, and sensitivity analyses | - |
| Discussion | | | |
| Key results | 18 | Summarise key results with reference to study objectives | 11 |
| Limitations | 19 | Discuss limitations of the study, taking into account sources of potential bias or imprecision. Discuss both direction and magnitude of any potential bias | 14 |
| Interpretation | 20 | Give a cautious overall interpretation of results considering objectives, limitations, multiplicity of analyses, results from similar studies, and other relevant evidence | 12-13 |
| Generalisability | 21 | Discuss the generalisability (external validity) of the study results | 12-14 |
| Other information | | | |
| Funding | 22 | Give the source of funding and the role of the funders for the present study and, if applicable, for the original study on which the present article is based | 15 |

*Give information separately for exposed and unexposed groups.

**Note:** An Explanation and Elaboration article discusses each checklist item and gives methodological background and published examples of transparent reporting. The STROBE checklist is best used in conjunction with this article (freely available on the Web sites of PLoS Medicine at http://www.plosmedicine.org/, Annals of Internal Medicine at http://www.annals.org/, and Epidemiology at http://www.epidem.com/). Information on the STROBE Initiative is available at http://www.strobe-statement.org.

**References.**

1. Pinero J, Ramirez-Anguita JM, Sauch-Pitarch J, et al. The DisGeNET knowledge platform for disease genomics: 2019 update. *Nucleic Acids Res.* 2020;48(D1):D845-D855.

2. Dweep H, Gretz N. miRWalk2.0: a comprehensive atlas of microRNA-target interactions. *Nat Methods.* 2015;12(8):697.
